# In search of an optimum sampling algorithm for prediction of soil properties from infrared spectra

**DOI:** 10.7717/peerj.5722

**Published:** 2018-10-03

**Authors:** Wartini Ng, Budiman Minasny, Brendan Malone, Patrick Filippi

**Affiliations:** Faculty of Science: School of Life and Environmental Sciences, University of Sydney, Sydney, New South Wales, Australia

**Keywords:** Calibration sample size, Infrared spectroscopy, Sampling algorithms, Soil properties, Regression

## Abstract

**Background:**

The use of visible-near infrared (vis-NIR) spectroscopy for rapid soil characterisation has gained a lot of interest in recent times. Soil spectra absorbance from the visible-infrared range can be calibrated using regression models to predict a set of soil properties. The accuracy of these regression models relies heavily on the calibration set. The optimum sample size and the overall sample representativeness of the dataset could further improve the model performance. However, there is no guideline on which sampling method should be used under different size of datasets.

**Methods:**

Here, we show different sampling algorithms performed differently under different data size and different regression models (Cubist regression tree and Partial Least Square Regression (PLSR)). We analysed the effect of three sampling algorithms: Kennard-Stone (KS), conditioned Latin Hypercube Sampling (cLHS) and k-means clustering (KM) against random sampling on the prediction of up to five different soil properties (sand, clay, carbon content, cation exchange capacity and pH) on three datasets. These datasets have different coverages: a European continental dataset (LUCAS, *n* = 5,639), a regional dataset from Australia (Geeves, *n* = 379), and a local dataset from New South Wales, Australia (Hillston, *n* = 384). Calibration sample sizes ranging from 50 to 3,000 were derived and tested for the continental dataset; and from 50 to 200 samples for the regional and local datasets.

**Results:**

Overall, the PLSR gives a better prediction in comparison to the Cubist model for the prediction of various soil properties. It is also less prone to the choice of sampling algorithm. The KM algorithm is more representative in the larger dataset up to a certain calibration sample size. The KS algorithm appears to be more efficient (as compared to random sampling) in small datasets; however, the prediction performance varied a lot between soil properties. The cLHS sampling algorithm is the most robust sampling method for multiple soil properties regardless of the sample size.

**Discussion:**

Our results suggested that the optimum calibration sample size relied on how much generalization the model had to create. The use of the sampling algorithm is beneficial for larger datasets than smaller datasets where only small improvements can be made. KM is suitable for large datasets, KS is efficient in small datasets but results can be variable, while cLHS is less affected by sample size.

## Introduction

In the last few decades, there has been growing interest in rapid soil characterisation. Infrared spectroscopy has gained interest for various soil analyses over the conventional ‘wet chemistry’ methods because the latter is laborious, costly and time-consuming. Furthermore, multiple soil properties can be predicted from a single soil spectrum (Bendor & Banin, 1995; Stenberg et al., 2010; Viscarra Rossel et al., 2008). Although spectroscopy utilizes wide ranges of the electromagnetic spectrum, the work presented in this study focuses on the visible near infrared (vis-NIR) region. The vis-NIR instrument allows a robust analysis of soil in the field or lab with little to no sample preparation.

In the mid-infrared region (MIR), the absorption is due to fundamental vibrations of organic and inorganic molecules in the soil; while in the vis-NIR region, absorption is due to overtones and the combinations of the fundamental vibrations found in the MIR region (Viscarra Rossel et al., 2008). Although the absorbance in the vis-NIR region is often broad and less resolved, this region contains some useful information on stretching and bending of the fundamentals C-H, N-H, O-H, and C=O bonds. With the help of chemometric techniques, properties of a soil sample can be predicted from its spectral absorption based on a regression model. The regression model is calibrated from a spectral library, relating infrared absorbance to standard laboratory measurements. The most common calibration models for soil applications are based on linear regressions, such as principal component regression (Chang et al., 2001; Stenberg et al., 2010) and partial least squares regression (PLSR) (McCarty et al., 2002; Wold, Johansson & Cocchi, 1993). Nonetheless, because soil is a complex medium that might have non-linear reflectance behaviour, a linear modelling approach like PLSR might not be sufficient (Vohland et al., 2011). Machine learning regression models, such as Cubist regression tree (Quinlan, 1993), random forests (RF) (Breiman, 2001), artificial neural networks (ANN) (Haykin, 1998) and support vector machines (SVM) (Vapnik, 2000) have been explored for its potential ability to yield higher accuracies.

Spectroscopy in conjunction with these chemometric techniques have been proven to predict various chemical and physical properties of soil, such as pH, cation exchange capacity (CEC), carbonate content, organic carbon content, and soil texture (Bendor & Banin, 1995; Chang et al., 2001; Islam, Singh & McBratney, 2003; Shepherd & Walsh, 2002). Nonetheless, the accuracy of these regression models to produce accurate predictions relies heavily on the calibration dataset used. To obtain a reliable prediction, representative data should be used in the model (Kuang & Mouazen, 2012; Viscarra Rossel et al., 2008). The number of calibration samples also affects the model predictions, although this has received limited attention (Kuang & Mouazen, 2012). A larger calibration sample size may be able to create more reliable and representative models compared to those models based upon smaller sample sizes (Kuang & Mouazen, 2012). However, in a real-world situation, the number of samples (with complete standard measurements) are usually small due to budget and/or time constraints (Minasny & McBratney, 2006). The optimal sample size is often determined by the balance between the budget and acceptable accuracy. With the expensive cost of soil analysis and limited budgets, choosing representative samples for laboratory analysis which are subsequently used for calibration, is a critical component in ensuring the establishment of the most appropriate regression models (Brown, Bricklemyer & Miller, 2005; Ramirez-Lopez et al., 2014).

**Figure 1:**
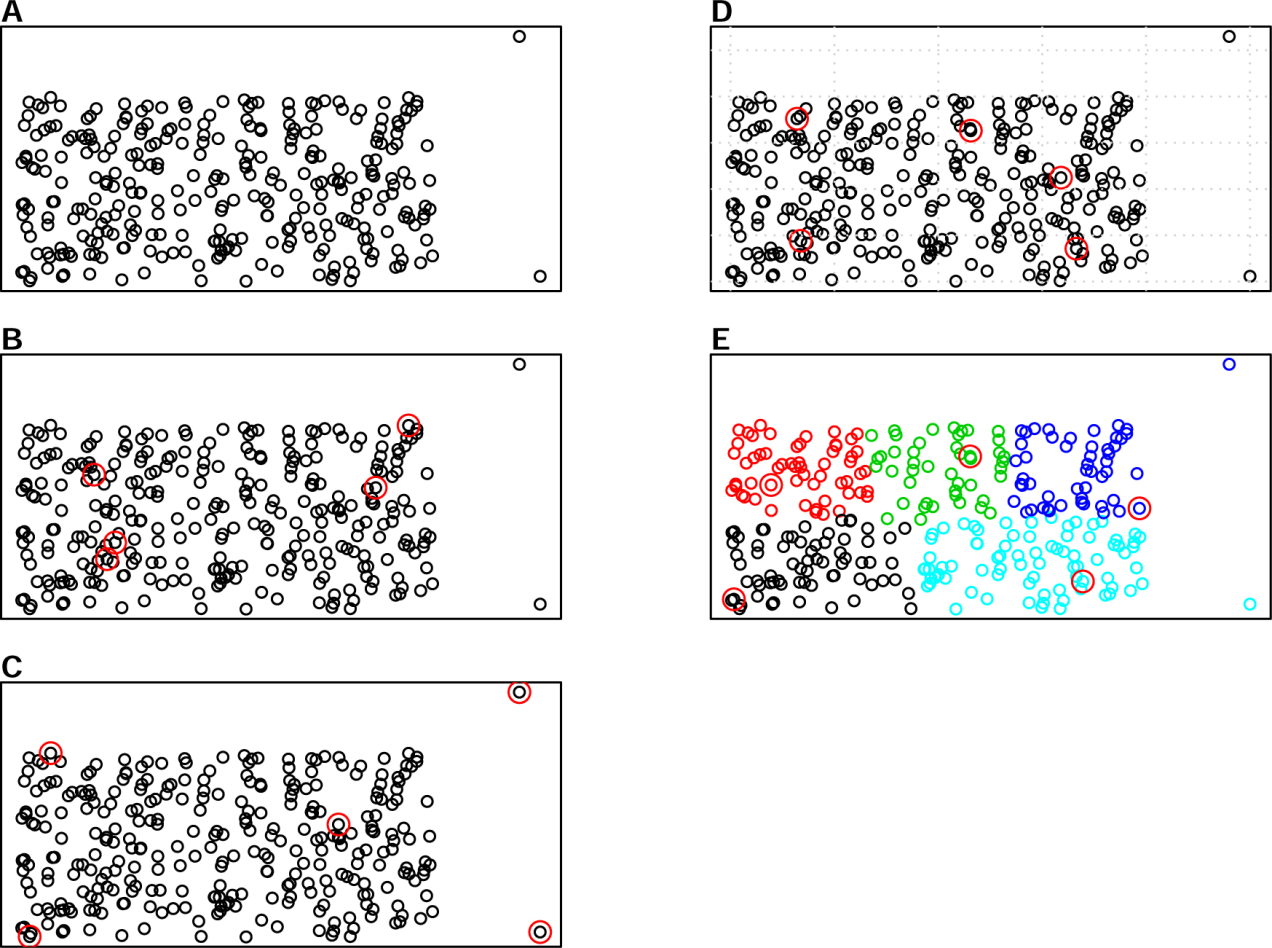
Illustrations of the various sampling strategies with sample population containing outliers: selecting 5 samples out of the 300 sample population. The red circles represent the samples selected by a particular sampling algorithm. (A) represents sample population, (B) represents random sampling, (C) represents the Kennard- Stone (KS) algorithm, (D) represents the conditioned Latin Hypercube sampling (cLHS) algorithm (E) represents the k-means clustering algorithm (KM).

There are various sampling algorithms available to select calibration samples in infrared spectroscopy, such as the Kennard-Stone (KS) algorithm, the conditioned Latin Hypercube Sampling (cLHS) and k-means clustering (KM). One of the most common sampling algorithms used in the infrared spectroscopy literature is the KS algorithm (Ramirez-Lopez et al., 2014), which sequentially selects samples with the largest distance in the variable space in the calibration set (Kennard & Stone, 1969). The cLHS algorithm developed initially for generating optimal sample configurations for digital soil mapping has also been used in soil spectroscopy studies (Mulder, De Bruin & Schaepman, 2013). The cLHS algorithm selects samples that optimally represent the multivariate distribution of the input dataset. The KM algorithm, on the other hand, partitions data into groups (strata) that have similar properties. Random sampling is then used to pick representative samples from each strata. An illustration of the three sampling algorithms as well as random sampling is given in Fig. 1. Aside from the random sampling, these three algorithms are utilized to optimize the selection of representative samples from the sample population.

Ramirez-Lopez et al. (2014) compared the use of KS, cLHS and fuzzy k-means clustering sampling (FKM) to select the calibration samples, and found that although KS algorithm was outperformed by other algorithms in terms of sample representativeness, the predictive performance of regression models for the prediction of clay content and exchangeable Ca (Ca^2+^) were comparable regardless of the sampling method. This study warrants further research as it only considers two properties for a field (5 km^2^) and regional scale (<500 km^2^) with a calibration sample size of up to 380 samples for each dataset.

In this study, we compared three sampling algorithms (KS, cLHS, and KM) against random sampling on three different datasets at continental, regional, and local scale with various calibration sample sizes using two different regression methods: PLSR and Cubist regression modelling. The performance of the models is evaluated based on the average prediction accuracies of up to five different soil properties (sand, clay, carbon content, cation exchange capacity and pH). Thus, the objective of this paper is to investigate the effect of calibration sample size, the efficiency of sampling algorithms, and regression methods to predict various soil properties on soil samples from three different spatial extents.

## Materials and Methods

### Datasets description

Three datasets were used in this study. The first dataset is from Europe which represents a continental database. The second is a regional database from southern New South Wales (NSW) and northern Victoria (VIC), and the third is a local database from the locality of Hillston in south-west NSW, Australia.

#### Dataset 1: Continental dataset

Dataset 1 was obtained from the Land Use/Land Cover Area Frame Survey (LUCAS) database (European Commission, 2017). The LUCAS soil database was developed as an attempt to create a consistent spatial database across the European Union. The survey covers a range of landscapes, with area coverage of approximately 4.5 million square kilometers (km^2^). This database is a collection of composite soil samples from 0–20 cm depth. All samples were scanned with a FOSS CDS Rapid Content Analyzer (NIRSystems, INC.) operating within 400–2,500 nm wavelength range with 0.5 nm spectra resolution. Each spectrum is composed of 4,200 wavelengths. Only one-third of the database were considered for this study to reduce computational time, resulting in a subset of 5,639 observations. All samples had been analyzed for particle size distribution (clay and sand content), pH (in CaCl_2_), organic carbon (g/kg), and cation exchange capacity (CEC; cmol/kg) among all other properties.

#### Dataset 2: Regional dataset

Dataset 2 consists of 379 soil samples of 68 different soil profiles from the wheat-belt of southern NSW and northern VIC covering approximately a 5,000 km^2^ area (Geeves et al., 1995). There is a large variation of soil in the area, but the major soil types are Alfisols and Oxisols. The soil samples were collected at different horizons with depth up to 1 m. The samples were air-dried, ground and sieved through a 2-mm sieve. The reflectance spectra were then collected with an AgriSpec (Analytical Spectral Devices, Boulder, CO, USA) with a spectral range of 350 to 2,500 nm with 1 nm sampling interval. A Spectralon (Labsphere Inc., North Sutton, NH, USA) white standard was used for instrument calibration. Each spectrum consists of 2151 wavelengths. All samples had been analysed for the clay and sand content (%), pH in CaCl_2_ (1:5), total carbon (%) and CEC (cmol/kg).

#### Dataset 3: Local dataset

Dataset 3 consists of soil samples from different soil cores extracted to 1.5 m from the cotton-growing district of Hillston in south-west NSW (Filippi et al., 2018a). The study area is approximately 2,650 km^2^ in size. The samples were collected in a survey conducted in 2002, consisting of 384 samples from 87 different sites. The soils in this area are mainly Vertisols, with some soils of sandier texture derived from Aeolian parent material (Filippi et al., 2018b). The soil samples were air-dried, ground and passed through a 2-mm sieve. Samples were then scanned using AgriSpec (Analytical Spectral Devices, Boulder, CO, USA) with a spectral range of 350 to 2,500 nm with 1 nm sampling interval. A Spectralon (Labsphere Inc., North Sutton, NH, USA) white standard was used for instrument calibration. Each spectrum consists of 2,151 wavelengths. These samples had been analyzed for total carbon, clay and sand content (%), pH (in H_2_O), and CEC (cmol/kg) (Filippi et al., 2018a)

### Data pre-processing

**Figure 2:**
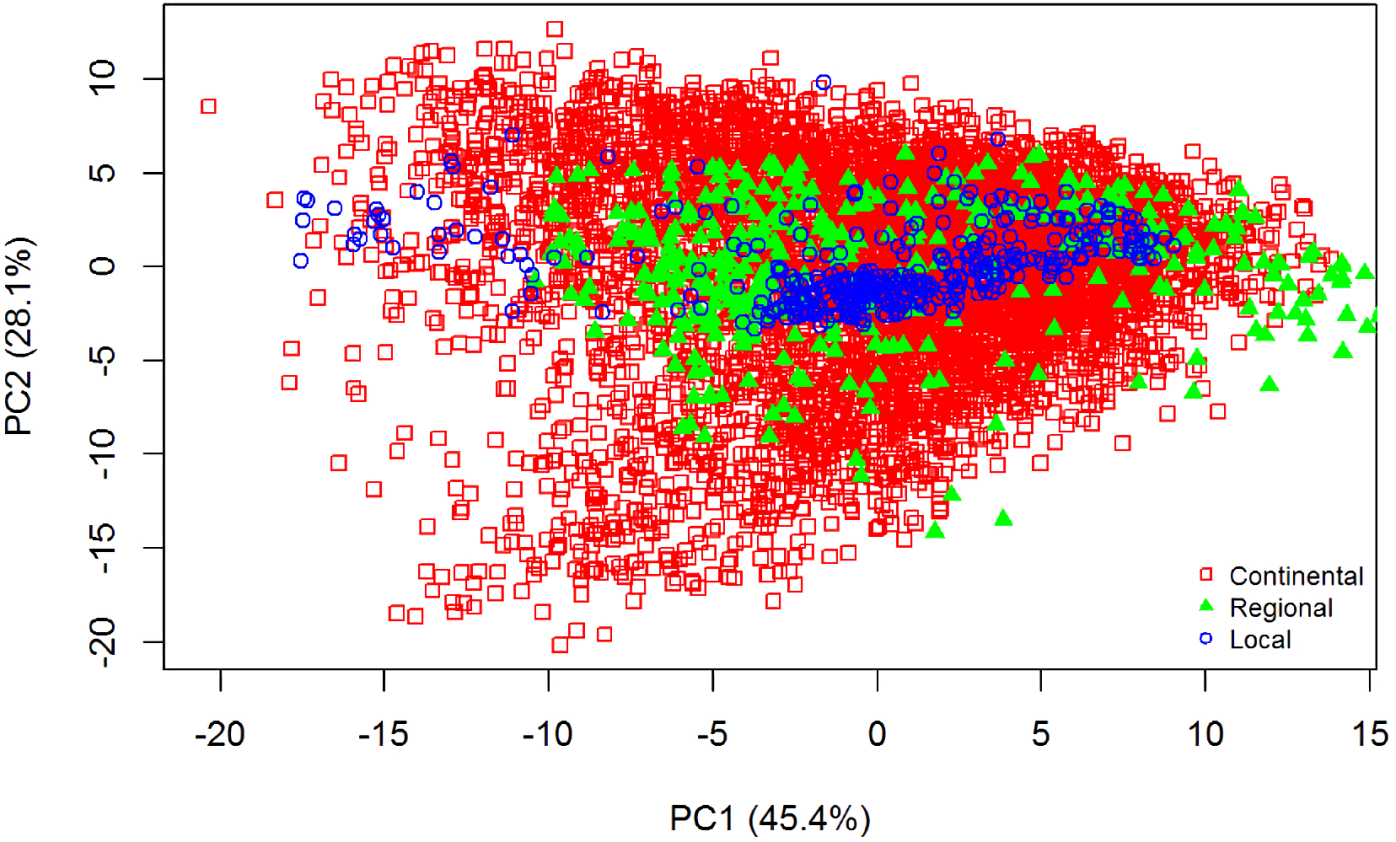
Principal Component Analysis (PCA) scores plot (PC1 vs. PC2) for visible near infrared (vis-NIR) spectra from the three different datasets: continental, regional and local. The PCA was performed on the pre-processed vis-NIR spectra.

The summary statistics for all datasets are included in Table 1. Data that were skewed, with a value greater than +2 or less than −2 (Curran, West & Finch, 1996), were subjected to natural log transformation to normalise the dataset. To explain the variability of the samples used for all three datasets, principal component analysis (PCA) of the pre-processed spectra was employed. The PCA distribution of the spectra of all the datasets is shown in Fig. 2. The differences of the three datasets is clearly shown; there is more variance in the continental dataset (LUCAS), followed by the regional dataset (Geeves), and less variance in the local dataset (Hillston).

**Table 1:** Summary statistics of soil properties in the datasets.

	Calibration set							Validation set						
	**Number of samples**	**Min.**	**Median**	**Mean**	**Max.**	**SD**	**Skewness**	**Number of samples**	**Min.**	**Median**	**Mean**	**Max.**	**SD**	**Skewness**
**Dataset1: continental**														
pH_CaCl2	3,639	2.66	5.89	5.79	9.25	1.34	−0.27	1,000	3.11	5.78	5.72	8.01	1.32	−0.2
CEC (cmol/kg)		0	11.8	13.87	78.5	9.55	1.31		0	11.35	13.87	59.9	9.96	1.36
Clay (%)		0	17	19.21	79	13.12	0.9		1	17	19.18	79	13.00	0.92
Sand (%)		1	41	42.35	98	26.05	0.23		1	42	41.91	98	26.01	0.19
Organic Carbon (g/kg)		0	18.9	24.96	99.5	18.67	1.63		0	19.5	25.6	99.5	18.78	1.49
**Dataset2: Regional**														
pH_CaCl2	284(51)^*^	3.84	5.31	5.43	8.03	0.89	0.6	95(17)^*^	3.76	5.45	5.7	8.23	1.17	0.53
CEC (cmol/kg)		0.4	7.08	8.62	28.21	5.12	1.15		1.6	8.87	10.88	36.43	7.21	1.33
Clay (%)		5	20	26.06	70	16.23	1		7	21	29.09	74	17.28	0.96
Sand (%)		14	60	57.12	91	16.42	−0.46		17	59	55.82	81	16.47	−0.7
Total Carbon (%)		0.06	0.83	1.19	12.74	1.48	4.3		0.11	0.93	1.16	5.9	1.04	2.2
**Dataset3: local**														
pH	298(66)^*^	5.8	8.83	8.61	10.06	0.86	−0.8	86(21)^*^	6.33	8.87	8.68	9.92	0.85	−0.82
CEC (cmol/kg)		3.19	28.67	26.88	50.71	9.18	0.76		2.65	27.89	26.84	53.84	9.04	−0.41
Clay (%)		8.7	53.7	49.47	64.4	12.56	−1.79		4.4	51.85	46.9	63.7	13.19	−1.51
Sand (%)		19.73	35.55	39.28	90.26	13.97	1.98		23.81	38.41	42.21	94.73	13.53	1.7

**Notes.**

* The number in parentheses represents the number of different sites where the samples originated from.

### Spectra pre-processing

**Figure 3:**
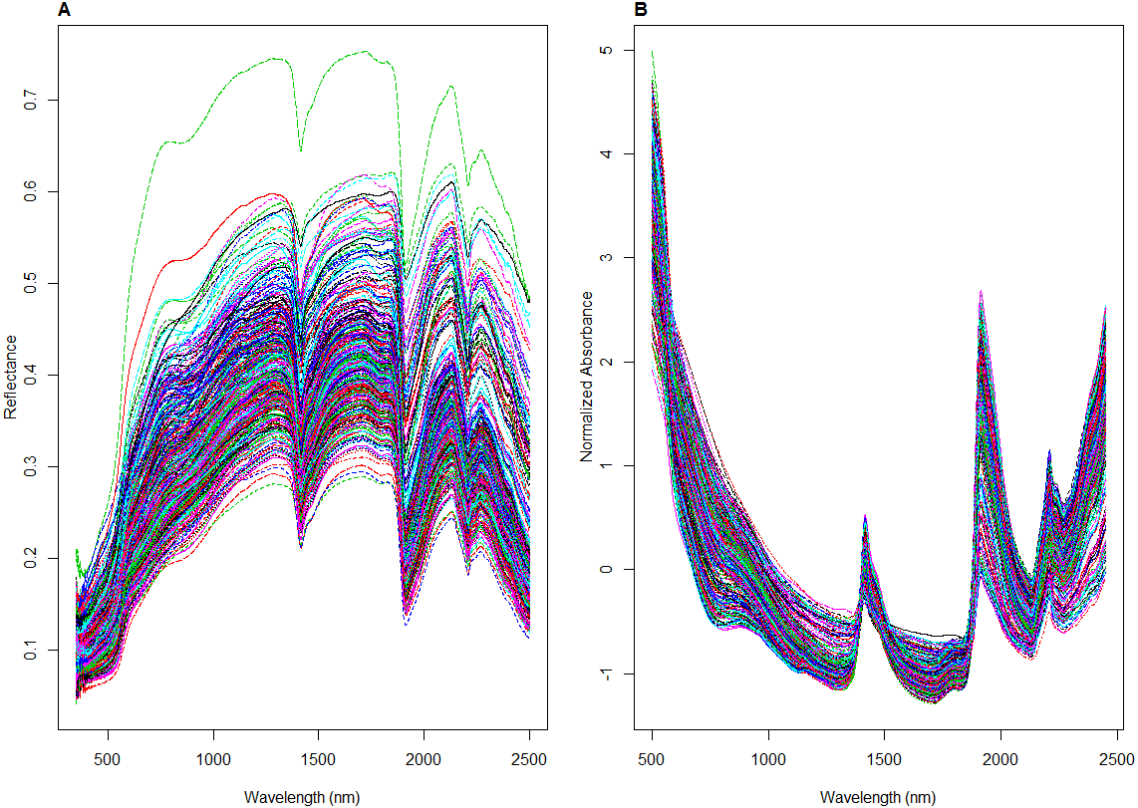
Illustrations of visible near infrared (vis-NIR) spectra from the local dataset: (A) raw and (B) being pre-processed.

To ensure that all the spectra from the different datasets underwent the same spectra pre-processing treatment, spectra from the LUCAS dataset were resampled every 1 nm to have the same sampling intervals, resulting in 2,100 points. Spectra between 350–499 nm and 2,451–2,500 nm range were removed due to their low signal to noise ratio resulting in 1951 point spectra for all datasets. The resulting spectra were transformed to absorbance log (1/R), and pre-processed by Savitzky-Golay (SG) transformation (Savitzky & Golay, 1964) with a window size of 11 and polynomial order 2 and followed with the Standard Normal Variate (SNV) transformation. SG algorithm is used to remove instrument noise within the spectra by smoothing the data using the polynomial regression, while SNV is used to normalize the spectra, scaling it to zero mean and unit standard deviation (Rinnan, Van den Berg & Engelsen, 2009). An example of the spectra before and after pre-treatment is shown in Fig. 3.

### Sampling algorithms

Three different sampling algorithms were tested in this study against random sampling, including Kennard Stone (KS), conditioned Latin Hypercube Sampling (cLHS), and k-means clustering (KM). All of the sampling methods are based on different principles of selecting samples from the available spectra data to be used for model calibration. Except for the random sampling, the three other sampling algorithms were utilized to optimize the selection of representative samples from the spectra. Ideally, the samples selected to be used for model calibration should explain the variability in the original samples and ultimately provide reliable predictions on the validation dataset (Soriano-Disla et al., 2014).

#### Random sampling

This is the simplest way of selecting samples. It creates a subset that follows the statistical distribution of the original dataset. While this is an unbiased method, it is not efficient as more samples are required to achieve the representativeness of the data (Rajer-Kanduc, Zupan & Majcen, 2003; Wu et al., 1996). Despite this shortcoming, the method is still commonly used as it is easy to carry out, and unbiased. Shepherd & Walsh (2002), McCarty et al. (2002), and Okparanma & Mouazen (2013) are some exemplar studies where this sampling approach has been utilized in soil spectra modelling studies.

#### Kennard Stone Sampling (KS)

This algorithm was developed initially to create a response surface of experimental design (Kennard & Stone, 1969) by selecting subset samples that cover the maximum distances between each candidate samples. It is a sequential and deterministic procedure. Consider *k* samples have been selected, where *k* <number of samples (N) in the dataset. The next sample candidate (*k* +1) has the furthest distance (in variable space) from existing samples with the following criteria:

\begin{eqnarray*}d={\max \nolimits }_{{i}_{o}}({\mathrm{min}}_{i}({d}_{i,io})) \end{eqnarray*}

where *i* is the existing sample candidate, and *i*_o_ is the candidate sample to be chosen. Here, the Euclidean distance is used (Kennard & Stone, 1969). This method is the most commonly used in the spectroscopy literature (Bouveresse & Massart, 1996; Ji, Rossel & Shi, 2015), however its efficiency in selecting representative sample is not well studied.

#### Conditioned Latin Hypercube Sampling (cLHS)

Conditioned Latin Hypercube sampling has its origins in Latin Hypercube sampling (LHS), which was first proposed by Mckay, Beckman & Conover (1979). LHS is an efficient way to reproduce an empirical distribution function, where the idea is to divide the empirical distribution function of a variable, *X* (for soil spectral data this could be an individual wavelength), into *n* equi-probable, non-overlapping strata, and then draw one random value from each stratum. In a multi-dimensional setting (for example a full spectrum), for *k* variables, *X*_1_*,X*_2_*,…,X*_*k*_, the *n* random values drawn for variable *X*_1_ are combined randomly (or in some order to maintain its correlation) with the *n* random values drawn for variable *X*_2_, and so on until *n k*-tuples are formed, i.e., the Latin hypercube sample (Clifford et al., 2014). Its utility for soil sampling was noted by Minasny & McBratney (2006), but they recognised that some generalisation of LHS sampling was required so that selected samples actually existed. Subsequently, they proposed a conditioning of the LHS, which is achieved by drawing an initial Latin hypercube sample from the ancillary information, then using simulated annealing to permute the sample in such a way that an objective function is minimised. The method was originally developed to select samples for calibration in digital soil mapping studies. Viscarra Rossel et al. (2008) adapted this sampling scheme to select representative samples from the legacy dataset to be sent for laboratory analysis.

#### K-means cluster sampling (KM)

K-means is a method to group data that are similar to each other into clusters. First, the data are allocated to the pre-defined number of centroids (center of the clusters). It is then optimized by minimizing the distance between the values of the data to its designated centroid while maximizing the distances among all the centroids. In this case, we utilized the Euclidean distance. Each data is reassigned to a cluster with the nearest centroid, and the new means becomes the new centroids. This process continues until no change in cluster members are observed (Næs, 1987). Random sampling is then utilized to select sample from each cluster. This method had been used by McDowell et al. (2012) to cluster samples to be included in the calibration dataset.

### Establishment of calibration models

All spectra derivation and calculation were performed with R statistical language and open-source software (R Core Team, 2016). For each sampling design, the predictive ability of different calibration sampling sizes were evaluated as the average of fifty repetitions of overall root mean square error (RMSE) and *R*^2^ values for the prediction of the various soil properties on the validation dataset. Other accuracy parameters (bias and RPIQ) are included in the Supplementary Material.

Each of the dataset was first randomly split into calibration and validation set (∼75% and ∼25% respectively). For the continental dataset, 1,000 samples were retained as the validation set, and the rest of the samples were utilized as a calibration set. In the smaller datasets (regional and local), the topsoil and subsoil samples were paired prior to data splitting. The dataset were split based on the unique profile location as suggested by Brown, Bricklemyer & Miller (2005) (see Table 1). This method is selected to ensure that the regression model can generalize based on the calibration dataset to predict on the validation dataset because the sample size is relatively small. Samples from 17 different sites with a total sample of 95 were used as validation in regional dataset. Meanwhile, 86 different samples from 21 different sites were used for validation in the local dataset.

To reduce the computational time, all the sampling strategies were applied to the principal components (PC) space of the pre-processed vis-NIR spectra. First, the principal component analysis was performed on all the dataset to determine how many principal components to be kept to explain 99% of the variances within the dataset. Nine, six and five PCs were retained for continental, regional and local dataset respectively. The R package ‘base’ was used to select the random samples (R Core Team, 2016), ‘prospectr’ to select the KS samples (Stevens & Ramirez-Lopez, 2013), ‘clhs’ to select the cLHS samples (Roudier, 2011), and ‘stats’ to select the KM samples (R Core Team, 2016).

**Figure 4:**
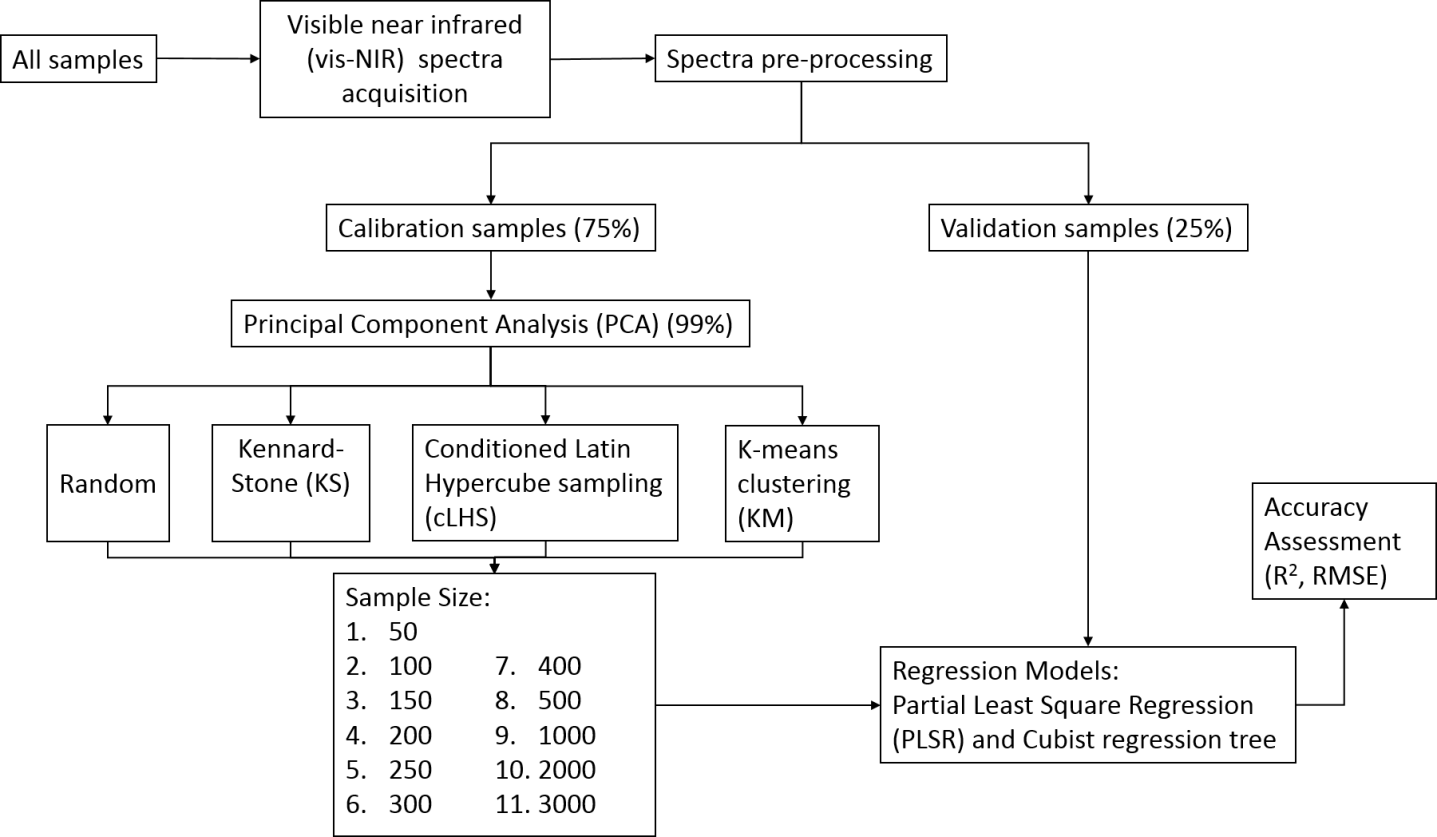
Methodology flowchart for creating various sizes calibration and validation.

The number of sample sizes was set at 50, 100, 150, 200, 250, 300, 400, 500, 1,000, 2,000 and 3,000 for the continental dataset, and 50, 100, 150 and 200 samples for both the regional and local dataset. All these different size calibration dataset models were validated with the same validation set from its respective dataset. All but the KS sampling algorithm were repeated fifty times and the average performances were reported in this study because the same samples were produced at each iteration, and hence removing the need of multiple repetitions. The methodology flow chart is illustrated in Fig. 4.

For each calibration set, the modelling required using R implementations of PLSR (Mevik, Wehrens & Liland, 2016) and Cubist models (Kuhn et al., 2016). PLSR is a linear chemometric regression model that projects spectra data into latent variables that explain the variances within the spectra data. The optimum number of components retained in the model corresponded to the number that provided the lowest cross-validation root means squared error of prediction (RMSEP). Cubist is a rule-based regression model developed by Quinlan (1993). If the input variables satisfy the regression rules, it is then passed into the multivariate linear regression models behind the rules instances. The Cubist model is run with the default hyperparameter settings. Hyperparameters are defined as parameters that have to be fixed before the running the model training (Probst, Bischl & Boulesteix, 2018), such as the number of committees, neighbours, and rules.

## Results

### Prediction of soil properties and effect of regression models

To investigate the effect of different types of regression models on prediction accuracy, the two models (PLSR and Cubist) were generated for each soil property and different calibration sample size for each dataset. This results in more than three thousand realizations and models for each dataset. The performance of the PLSR and Cubist regression model was evaluated on five soil properties for the continental and regional dataset and four soil properties for the local dataset. All results presented here are based on the validation set.

**Figure 5:**
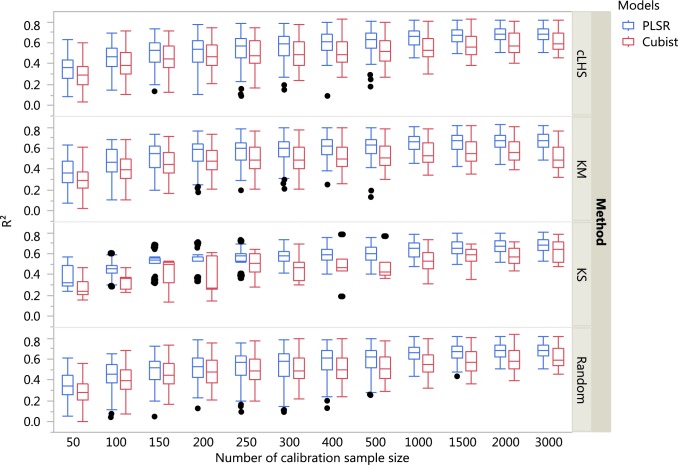
Boxplots comparing the performance of Partial Least Square Regression (PLSR) and Cubist regression tree models in predicting soil properties using various calibration sampling size and sampling algorithms within the continental dataset. Each boxplot represents the results for the 50 repetitions of the various soil properties predicted. cLHS, conditioned Latin Hypercube sampling; KM, k-means clustering; KS, Kennard-Stone.

**Figure 6:**
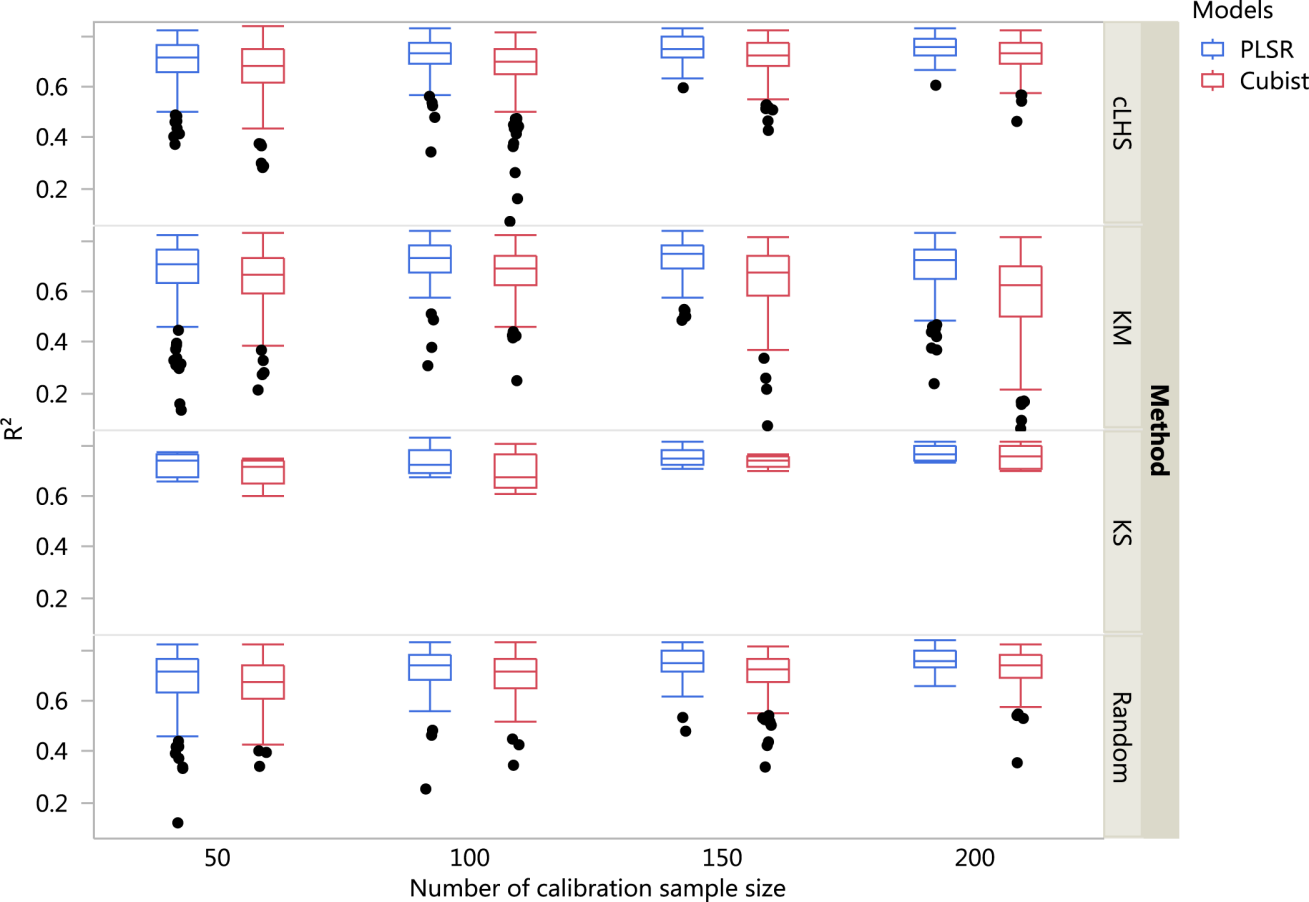
Boxplots comparing the performance of Partial Least Square Regression (PLSR) and Cubist regression tree models in predicting soil properties using various calibration sampling size and sampling algorithms within the regional dataset. Each boxplot represents the results for the 50 repetitions of the various soil properties predicted. cLHS, conditioned Latin Hypercube sampling; KM, k-means clustering; KS, Kennard-Stone.

**Figure 7:**
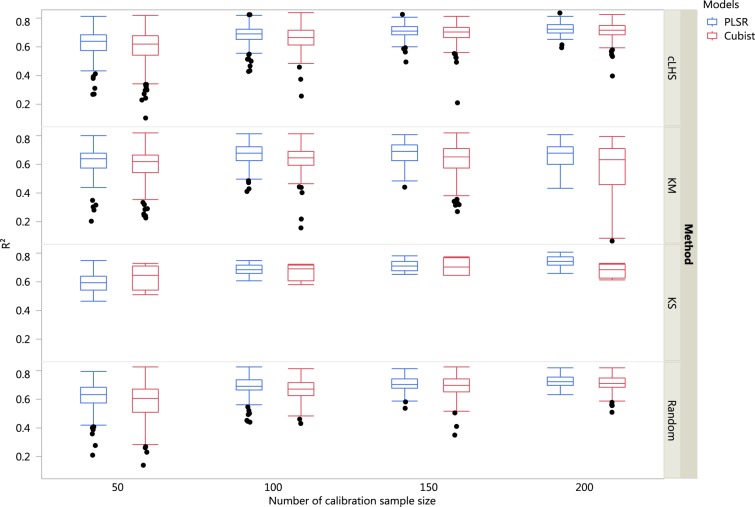
Boxplots comparing the performance of Partial Least Square Regression (PLSR) and Cubist regression tree models in predicting soil properties using various calibration sampling size and sampling algorithms within the local dataset. Each boxplot represents the results for the 50 repetitions of the various soil properties predicted. cLHS, conditioned Latin Hypercube sampling; KM, k-means clustering; KS, Kennard-Stone.

The boxplots comparing the two regression models (PLSR and Cubist) using various sampling algorithms with various calibration sample sizes for the different datasets are included in Figs. 5– 7. Each boxplot represents the average *R*^2^ value of various properties for that dataset using a given calibration sample size and sampling algorithm. For a comparison between the effects of regression models, only the performance of random sampling method is discussed in this section. The effect of sampling algorithm will be discussed later in the paper.

For the continental dataset, pH was predicted best using the PLSR model with calibration sample size of 3,000 (*R*^2^ = 0.81), followed by clay content (*R*^2^ = 0.73), CEC (*R*^2^ = 0.68), OC (*R*^2^ = 0.59) and sand content (*R*^2^ = 0.53). For the Cubist modelling and calibration sample size of 3,000, the model performance for each of the soil properties were: pH (*R*^2^ = 0.83), clay content (*R*^2^ = 0.70), CEC (*R*^2^ = 0.61), OC (*R*^2^ = 0.58) and sand content (*R*^2^ = 0.52). More detailed results are included in the Supplemental Information.

For the regional dataset with the calibration sample size of 200, using the PLSR model the ranking from the highest to lowest in terms of the *R*^2^ was CEC (*R*^2^ = 0.82), pH (*R*^2^ = 0.79), clay (*R*^2^ = 0.75), sand content (*R*^2^ = 0.74) and total C (*R*^2^ = 0.72). Using the Cubist model and calibration sample size of 200, the best performance of the model in terms of *R*^2^ were CEC (*R*^2^ = 0.80), pH (*R*^2^ = 0.73), clay content (*R*^2^ = 0.72), sand content (*R*^2^ = 0.71) and total carbon respectively (*R*^2^ = 0.70).

For the local dataset with the calibration sample size of 200, using the PLSR model the best models in terms of *R*^2^ were ranked as clay (*R*^2^ = 0.77), pH (*R*^2^ = 0.72), CEC (*R*^2^ = 0.71) and sand content (*R*^2^ = 0.70). With the Cubist model and calibration sample size of 200, the best-fitted models were clay (*R*^2^ = 0.73), followed by pH (*R*^2^ = 0.72), CEC (*R*^2^ = 0.69) and sand content (*R*^2^ = 0.68).

**Figure 8:**
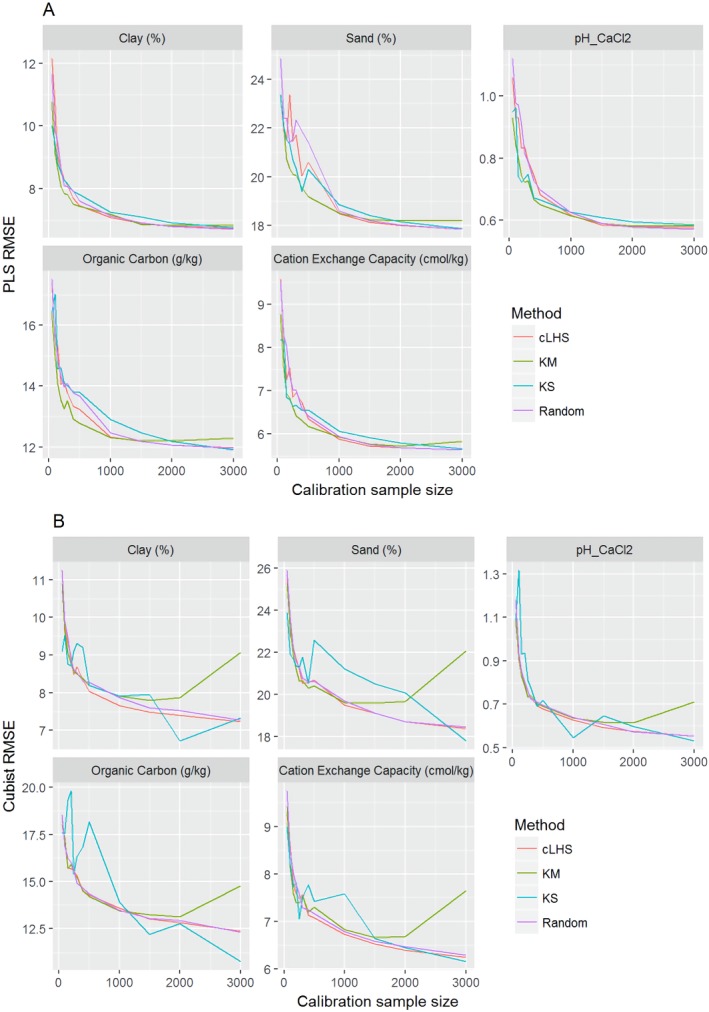
Plot of root mean square error (RMSE) against the number of calibration sample size for the prediction of various soil properties using: (A) Partial Least Square Regression (PLSR) and (B) Cubist tree regression models within the continental dataset. cLHS, conditioned Latin Hypercube sampling; KM, k-means clustering; KS, Kennard-Stone.

In general, the PLSR provided better prediction than the Cubist regression, regardless of the calibration sampling size and sampling algorithm (see Figs. 5– 7). The PLSR was also not heavily affected by the sampling algorithm in comparison to the Cubist regression. This effect was prominent in continental dataset as a more extensive sequence of calibration sample sizes were evaluated (see Fig. 8).

**Figure 9:**
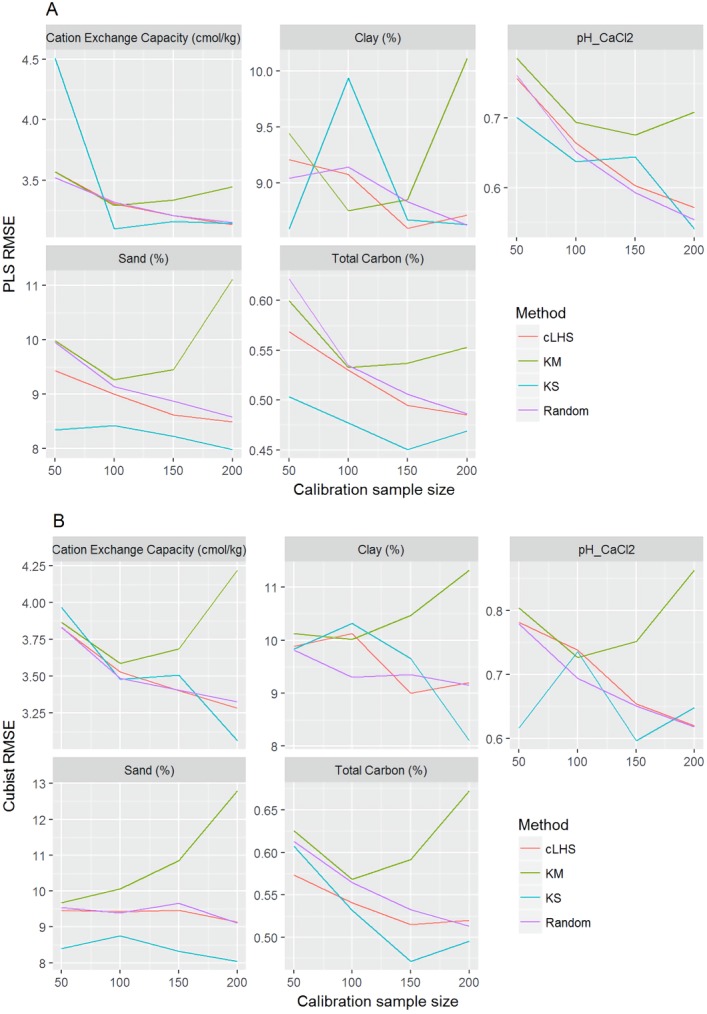
Plot of root mean square error (RMSE) against the number of calibration sample size for the prediction of various soil properties using: (A) Partial Least Square Regression (PLSR) and (B) Cubist tree regression models within the regional dataset. cLHS, conditioned Latin Hypercube sampling; KM, k-means clustering; KS, Kennard-Stone.

**Figure 10:**
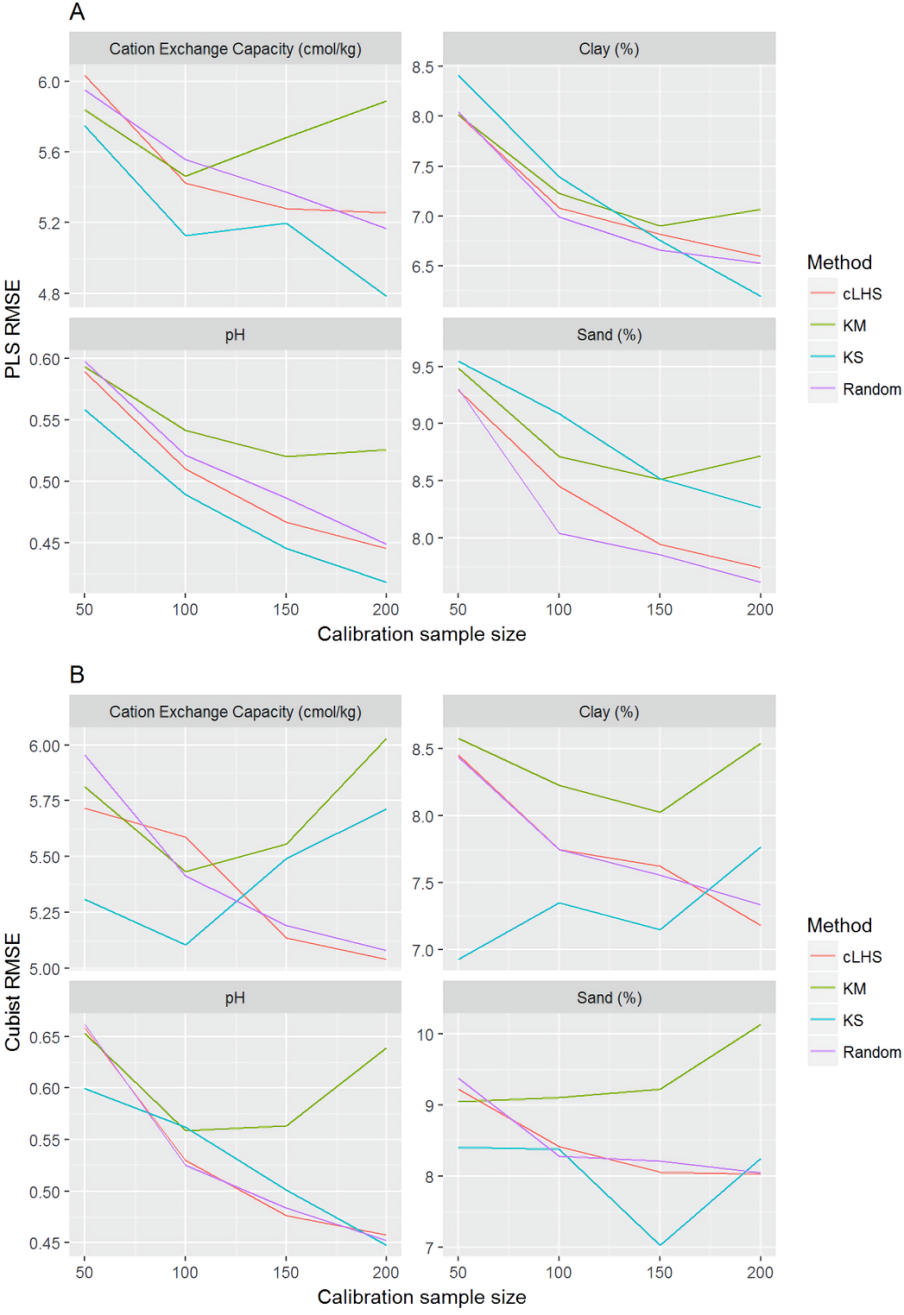
Plot of root mean square error (RMSE) against the number of calibration sample size for the prediction of various soil properties using: (A) Partial Least Square Regression (PLSR) and (B) Cubist tree regression models within the local dataset. cLHS, conditioned Latin Hypercube sampling; KM, k-means clustering; KS, Kennard-Stone.

In the continental dataset using the PLSR model, there was a steady increase in model performance (lower RMSE) as calibration sample size increased (see Fig. 8). All sampling algorithms behaved similarly. Meanwhile, the performance of the Cubist regression fluctuated depending on the sampling algorithm (see Fig. 8). For smaller calibration sample size in the smaller datasets, the KS algorithm provided the best performance (see Figs. 9 and 10). However, its performance was inconsistent. Since the KS algorithm tends to pick samples that explained the most variance, it tends to pick up the outlier/extreme samples. Combining the KS algorithm sample selection with rule-based algorithms such as the Cubist model could potentially lead to larger variance of the regression model. It was also noted that although the combined use of the KM algorithm and Cubist provided an overall good prediction, as the calibration sample size >2,000, the performance started to deteriorate. Regardless of the datasets and sample size, the performance of subset samples selected using cLHS mimics those of random sampling.

### The effect of calibration sample size

As the number of samples for calibration increased, the prediction became more accurate following the general pattern of a learning curve (see Fig. 8). The larger the calibration sample size dataset, the lower the RMSE validation was. These results are consistent with findings from other studies (Brown, Bricklemyer & Miller, 2005; Kuang & Mouazen, 2012; Ramirez-Lopez et al., 2014; Shepherd & Walsh, 2002).

Regardless of the sampling algorithm, the use of the PLSR model for the continental dataset, yielded pretty much similar performance with sample sizes greater than 1,000 (Fig. 8). By increasing the sample size from 500 to 1,000, the overall properties prediction improved an average of 6.4% (in terms of RMSE decrease). For calibration sample size 1,000 to 1,500, however, the improvement was only minimal at an average of 1.6%. This result is different to those of the findings from (Ramirez-Lopez et al. (2014)) where at calibration sample sizes ≥200, they observed that the error already stabilized. This is most likely due to much larger area coverage of the dataset used in this study. Meanwhile, for the smaller regional and local datasets, the calibration sample size results were inconclusive because no plateau had been reached at a sample size of 200. Although at calibration sample size of 200, the regression performance was good. This suggests that the regression could be further improved by increasing the size of the calibration sample (see Figs. 9A and 10A).

With the Cubist model in the continental dataset, the cLHS and KS algorithm converged to the performance of the random sampling at a sample size of 2,000. However, when using the KM algorithm, the predictions became worse with an increasing number of samples. No plateau had been reached in the smaller datasets (regional and local) using the Cubist model, with the KM algorithm performing worse as the calibration sample size increased. This means that for a large number of samples, the KM algorithm does not partition the data effectively, and should not be used.

The performance of the KS algorithm increased as sample size increased in the regional dataset, except for the prediction of pH. In the local dataset, only the pH prediction improved as calibration sample size increased to 200 (Figs. 9B–10B).

### The efficiency of the sampling algorithm

Firstly, we evaluate the sampling algorithm that produced the lowest error. For the continental dataset with the PLSR model, overall the KM algorithm performed best for clay, sand, pH and organic carbon (giving the lowest RMSE) for sample sizes <1,000 (Fig. 8A). The KS performed best for CEC at sample size <300. For the regional dataset with the PLSR model, the KS method performed best for all sample size and all properties, while the KM algorithm was the worst performing (Fig. 9A). The cLHS and random sampling appeared to perform similarly. For the local dataset, KS performed best for CEC and pH, while cLHS and random sampling performed best for sand and clay content (Fig. 10A).

**Figure 11:**
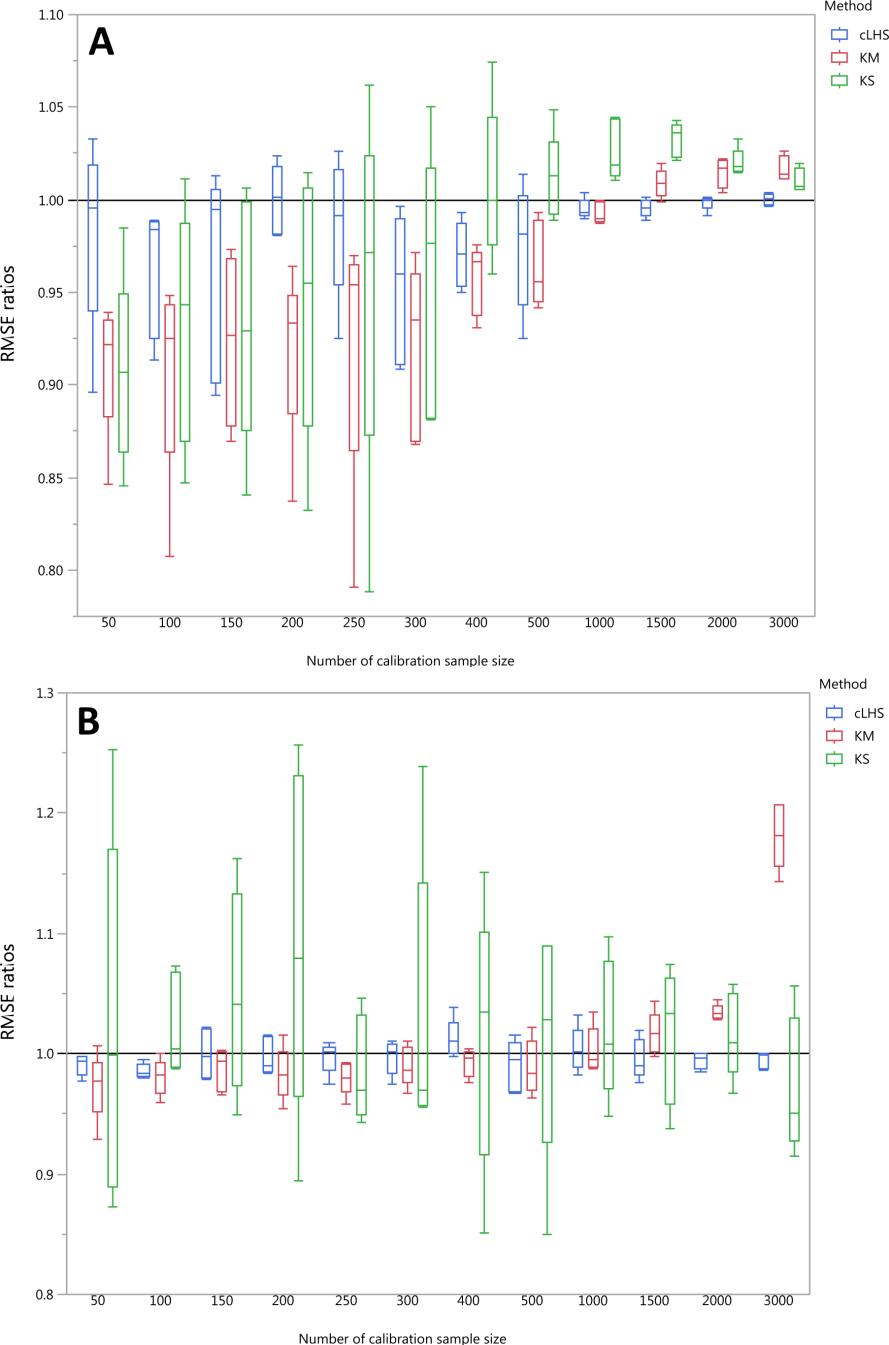
Average performances of various sampling algorithms for the prediction of five different soil properties using various calibration sample size in the continental dataset in terms of RMSE ratios using (A) Partial Least Square Regression (PLSR) and (B) Cubist model. Each boxplot represents the average of 50 repetitions of the five different soil properties predicted. The solid black line represents the average performance of the random sampling. cLHS, conditioned Latin Hypercube sampling; KM, k-means clustering; KS, Kennard-Stone.

**Figure 12:**
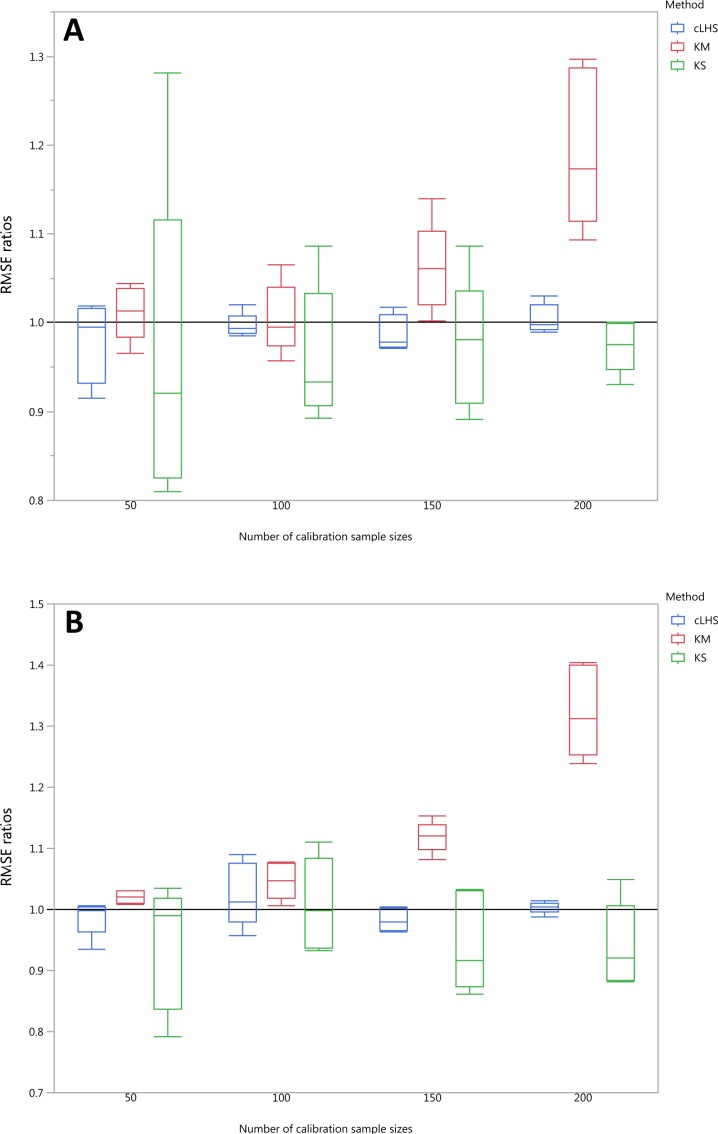
Average performances of various sampling algorithms for the prediction of five different soil properties using various calibration sample size in the regional dataset in terms of RMSE ratios using (A) Partial Least Square Regression (PLSR) and (B) Cubist model. Each boxplot represents the average of 50 repetitions of the five different soil properties predicted. The solid black line represents the average performance of the random sampling. cLHS, conditioned Latin Hypercube sampling; KM, k-means clustering; KS, Kennard-Stone.

**Figure 13:**
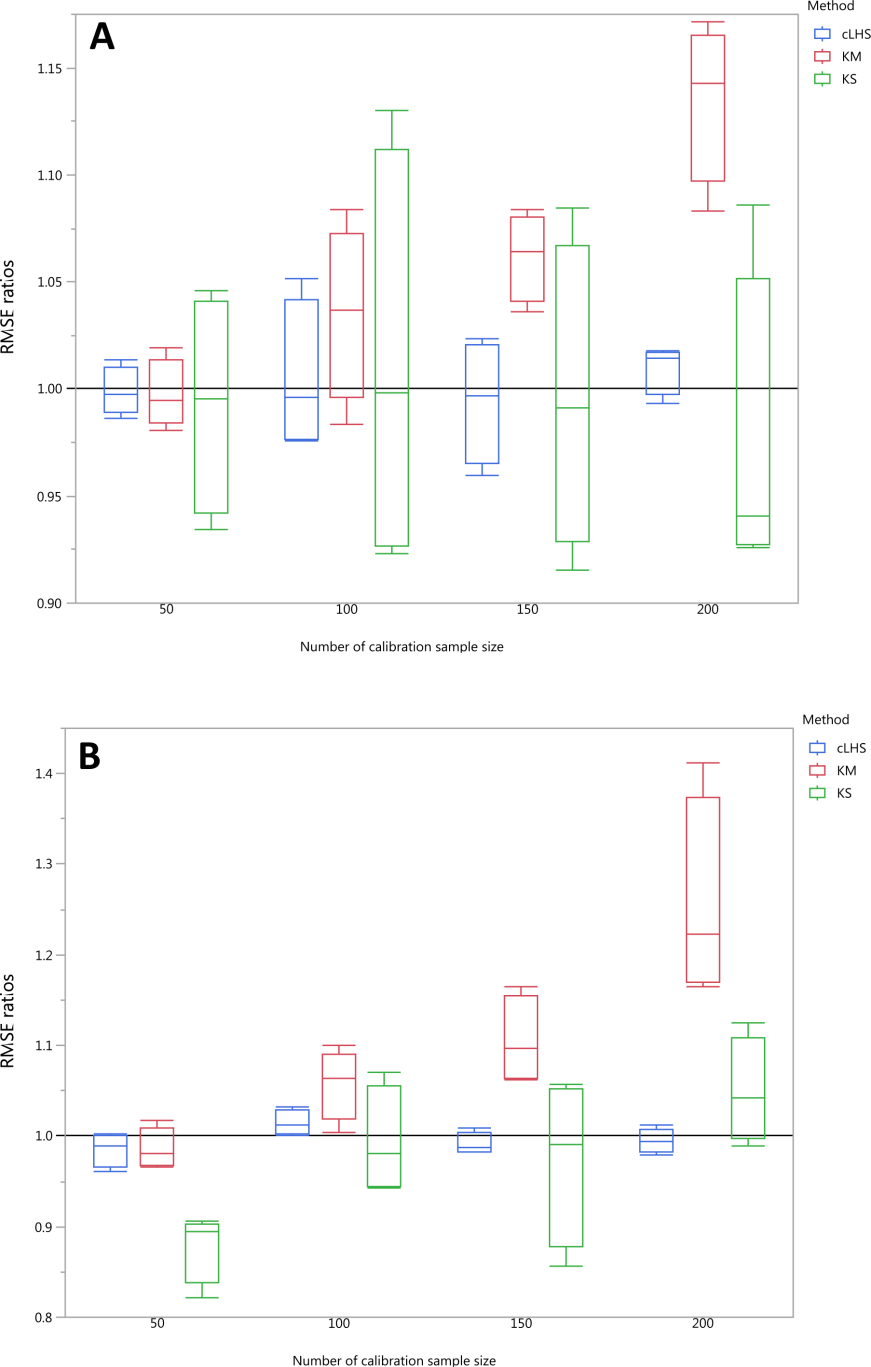
Average performances of various sampling algorithms for the prediction of four different soil properties using various calibration sample size in the local dataset in terms of RMSE ratios using (A) Partial Least Square Regression (PLSR) and (B) Cubist model. Each boxplot represents the average of 50 repetitions of the four different soil properties predicted. The solid black line represents the average performance of the random sampling. cLHS, conditioned Latin Hypercube sampling; KM, k-means clustering; KS, Kennard-Stone.

To be able to quantify the effectiveness of a sampling algorithm, its performance is compared against the performance of the random sampling method by way of the ratio between RMSE values from each sampling approach and the random sampling approach. The average performance prediction for the various soil properties were then plotted as boxplots illustrated in Figs. 11– 13 for the continental, regional and local dataset respectively. Each boxplot colour represents a particular sampling algorithm. The best sampling algorithm would have RMSE ratio <1, meaning it performed better than the random sampling.

For the continental dataset, the combination of an effective sampling algorithm with PLSR model could improve the overall model performance. The KS algorithm was able to provide a calibration subset dataset that improved the model performance in comparison to the random sampling up to sample size of 500 (see Fig. 11). For the calibration sample size greater than 500, the KS algorithm failed to perform better than the random sampling. The median RMSE reduction achieved with this algorithm was 0–10% (ranging from 0–15%). The model performance using calibration samples selected using the KM algorithm was able to provide similar calibration subset dataset up to sample size of 1500. Nonetheless, as sample size increased, the performance deteriorated and became worse than random sampling. Within the same sample size range of 500, the median RMSE reduction achieved by the KM was 4–8% (ranging from 1–15%). The samples selected using cLHS algorithm provided a much smaller RMSE reduction in comparison to the other two sampling algorithms with a median reduction in RMSE of 1–2% (ranging from 0–10%). On average, the samples selected using cLHS managed to perform better than those of random sampling up until calibration sample size of 3,000 where it performed similarly. The combinations of the sampling algorithms with the Cubist model yielded quite different results. The sample selected using KS algorithm in conjunction with the Cubist model yielded large variations in model performance, as pointed out earlier in the paper. Because of this, KS should be used with caution in conjunction with the Cubist model. The calibration subset data selected using KM algorithm performed worse than random sampling at a calibration sample size of 1,500. The calibration subset data selected using cLHS algorithm performed worse than random sampling at a sample size of 400; however, the performance improved and eventually became similar to that achieved when random sampling is used for sample data selection. Although calibration sample dataset selected using KM and cLHS sampling algorithm improved the Cubist model performance, this improvement was much less in comparison to the improvement observed in the PLSR model with RMSE improvement ranging from 0.86–2% and 0 – 1.4% respectively.

In the regional dataset, the KS algorithm with PLSR model performed best with a median RMSE reduction of 2–8% (ranging from 0–19%). The KM algorithm provided a subset of calibration dataset that contibuted to better model outcomes when compared to the random sampling, starting at calibration sample size of <150 (see Fig. 12). The cLHS algorithm provided samples with similar predictions as random sampling with minimal reduction in performance of 0.2–2.6% (ranging from 0–8.5%). The use of the KM algorithm with the Cubist model in the regional dataset failed to perform better than the random sampling. Similar to the observation in the continental dataset, the conjunction of KS algorithm and Cubist model yielded model performance with large variance. The average improvement achieved by the KS was a 2.5–7% reduction in RMSE (ranging from 0–20%). The RMSE improvement achieved with the cLHS algorithm was 0.25–1.8% (ranging from 0–6.5%).

In the local dataset, the KS algorithm also provided samples with the lowest RMSE prediction. However, note that the variation in the RMSE was quite large (see Fig. 13). The median RMSE reduction achieved was 1.6–6% (ranging from 0–8%). The KM algorithm performed worse than the random sampling starting at a calibration sample size of 100. cLHS consistently provided similar performance prediction as random sampling with RMSE reduction ranging from 0–4% with a median of 0–1%. With the Cubist model, the KM algorithm also deteriorated at a calibration sample size of 100. KS and cLHS algorithms in the Cubist behaved similarly to those in the PLSR model with minimal RMSE improvement using cLHS algorithm (median of 0.5–1% reduction in RMSE), and large variance in RMSE reduction using KS algorithm (median of 2.5–10.6%).

## Discussions

The choice of regression model clearly affected the model performance. In general, the PLSR model performed better than the Cubist model. This could be due to the un-optimized hyperparameters used in the Cubist model in this study. By adding number of committees or neighbours in the Cubist model, the model generated would be more robust. However, caution should be taken when tuning these hyperparameters as overfitting could be introduced when the calibration sample set is small.

Sample size and sample representativeness affected the performance of the regression model. As calibration sample size increased, the model performance improved which follows a pattern of a learning curve. Increasing sample size only could improve the model prediction up to a certain point, and further addition of calibration sample data would not lead to a better model. The optimum calibration sample size relied on how much generalization the model has to create. When the model performance is optimized, it is unnecessary to add more calibration samples.

Since the choice of sampling algorithm also affects the model performance, the selection thereof from a soil spectral modelling perspective requires due consideration. In particular, we found the combined use of regression models and a sampling algorithm that represents the sample population better (cLHS) have higher accuracy in comparison to those that tend to pick up the outlier in the sample population (KS), which logically makes sense. Although the KM algorithm performed well on the larger continental dataset and the KS algorithm performed best on the smaller regional and local datasets, the cLHS algorithm provided the most robust sampling algorithm. However, this efficiency of the sampling algorithm in improving predictions was more beneficial in the larger dataset. This suggests that sampling algorithms were not as effective in smaller datasets, and random sampling itself should be sufficient. Furthermore, the combined use of a sampling algorithm with certain regression models should be done with caution, as we showed earlier. The use of the KS algorithm in conjunction with Cubist models yielded large variations in model performance.

We noted that in this study, the sampling algorithms (cLHS, KM and KS) selected samples based on the principal components of the spectra, while the calibration models used the pre-processed spectra. Thus, their use in sampling algorithms may not be optimal, and perhaps that leads to the low performance of the cLHS method. Although similar results are expected, future research should look into comparing the performance of sampling algorithm both by using PCs as well as the pre-processed spectra.

## Conclusions

We explored the effect of three different sampling algorithms in comparison to random sampling on different calibration sample sizes using two different regression models on three different datasets.

• For the datasets we evaluated, generally, the PLSR model gives better performance in comparison to the Cubist model. It generated much more robust models regardless of the sampling algorithm. A future study could assess the optimization of Cubist hyperparameters.
• The Cubist tree model is highly affected by the choice of sampling algorithm, especially KS. The KS sampling technique is not recommended for use in rule-based or tree models.
• Although an increase in calibration set size could increase the performance of the model, we found that in a continental dataset, calibration sample size ≥1,000 does not provide much improvement to model prediction. This also means that only 25% of the samples need to be fully analysed to provide a good calibration set.
• The KM algorithm was suitable to select calibration dataset for larger datasets up to a point (∼1,000 samples), however, the performance deteriorated with increasing samples size, with KM being the worst for smaller datasets.
• Conversely, the KS algorithm performed better on the smaller datasets and worse in large datasets. As the algorithm picks extreme spectra, KS can result in a good calibration for certain soil properties, but poor calibration in other properties.
• The use of cLHS algorithm provided more robust sampling algorithms regardless of sample sizes.
• Overall, the efficiency of the sampling methods (in comparison to random sampling) is more significant in the larger dataset in comparison to the smaller datasets.

##  Supplemental Information

10.7717/peerj.5722/supp-1 Supplemental Information 1 Compiled results

10.7717/peerj.5722/supp-2 Supplemental Information 2 R code

## Additional Information and Declarations

### Data Availability

The following information was supplied regarding data availability: Ng, Wartini; Minasny, Budiman; Malone, Brendan; Filippi, Patrick (2018): Optimum sampling algorithm for the prediction of soil properties from the infrared spectra. figshare. Fileset. https://doi.org/10.6084/m9.figshare.6661856.v2.
