# Supplementary material for: In search of an optimum sampling algorithm for prediction of soil properties from infrared spectra
Source: PeerJ. 2018 Oct 3;6:e5722. doi: 10.7717/peerj.5722 (PMC6173947; doi:10.7717/peerj.5722)
Supplement: Supplemental Information 1 [file peerj-06-5722-s001.docx]

# Appendix A. Summary of model performance to predict various soil properties (clay content, sand content, pH, organic carbon and cation exchange capacity) using two different regression models (Partial Least Square Regression (PLSR) and Cubist) with various sampling algorithms (Random, Kennard-Stone (KS), conditioned Latin Hypercube sampling (cLHS), k-Means(KM)) and calibration sample sizes (50-3000) in the continental dataset. The results reported are averages and standard deviations from 50 repetitions.

| Sampling Algorithm | Calibration sample size | Soil Property | PLSR | | | | Cubist | | | |
| --- | --- | --- | --- | --- | --- | --- | --- | --- | --- | --- |
|  |  |  | R^2^ | RMSE | bias | RPIQ | R^2^ | RMSE | bias | RPIQ |
| Random | 50 | clay | 0.37 ± 0.08 | 11.64 ± 1.99 | 0.09 ± 1.34 | 0.70 ± 0.09 | 0.35 ± 0.10 | 11.25 ± 1.22 | -0.41 ± 1.64 | 0.72 ± 0.08 |
| KS | 50 | clay | 0.45* | 10.00* | 2.72* | 0.80* | 0.54* | 9.08* | -2.18* | 0.88* |
| cLHS | 50 | clay | 0.39 ± 0.10 | 12.15 ± 5.23 | -0.03 ± 1.22 | 0.71 ± 0.14 | 0.37 ± 0.10 | 11.24 ± 1.28 | -0.23 ± 1.84 | 0.72 ± 0.08 |
| KM | 50 | clay | 0.39 ± 0.08 | 10.75 ± 1.18 | 0.47 ± 1.49 | 0.75 ± 0.07 | 0.37 ± 0.09 | 10.88 ± 0.92 | -0.23 ± 1.72 | 0.74 ± 0.06 |
| Random | 100 | clay | 0.50 ± 0.08 | 9.84 ± 1.36 | 0.06 ± 1.07 | 0.83 ± 0.10 | 0.48 ± 0.09 | 9.91 ± 1.25 | -0.31 ± 1.11 | 0.82 ± 0.08 |
| KS | 100 | clay | 0.56* | 9.43* | 3.75* | 0.85* | 0.48* | 9.53* | -1.15* | 0.84* |
| cLHS | 100 | clay | 0.47 ± 0.11 | 10.67 ± 3.01 | 0.15 ± 0.67 | 0.79 ± 0.14 | 0.49 ± 0.07 | 9.77 ± 0.87 | -0.26 ± 0.92 | 0.83 ± 0.07 |
| KM | 100 | clay | 0.54 ± 0.08 | 9.12 ± 1.07 | 0.41 ± 0.93 | 0.89 ± 0.09 | 0.48 ± 0.08 | 9.65 ± 0.87 | -0.39 ± 1.12 | 0.84 ± 0.07 |
| Random | 150 | clay | 0.55 ± 0.08 | 9.47 ± 1.93 | 0.18 ± 0.95 | 0.87 ± 0.12 | 0.52 ± 0.09 | 9.48 ± 1.34 | 0.00 ± 1.05 | 0.86 ± 0.11 |
| KS | 150 | clay | 0.57* | 8.80* | 2.12* | 0.91* | 0.55* | 8.76* | -0.88* | 0.91* |
| cLHS | 150 | clay | 0.55 ± 0.07 | 9.02 ± 0.95 | 0.09 ± 0.57 | 0.90 ± 0.09 | 0.52 ± 0.07 | 9.32 ± 0.86 | -0.11 ± 0.79 | 0.87 ± 0.08 |
| KM | 150 | clay | 0.59 ± 0.08 | 8.60 ± 1.45 | 0.48 ± 0.72 | 0.95 ± 0.10 | 0.54 ± 0.08 | 9.07 ± 1.02 | 0.01 ± 0.93 | 0.89 ± 0.09 |
| Random | 200 | clay | 0.60 ± 0.07 | 8.65 ± 1.21 | 0.14 ± 0.74 | 0.94 ± 0.10 | 0.57 ± 0.09 | 8.85 ± 1.17 | -0.31 ± 0.86 | 0.92 ± 0.11 |
| KS | 200 | clay | 0.59* | 8.52* | 1.63* | 0.94* | 0.58* | 8.72* | -2.09* | 0.92* |
| cLHS | 200 | clay | 0.59 ± 0.07 | 8.63 ± 1.31 | 0.09 ± 0.58 | 0.94 ± 0.10 | 0.55 ± 0.07 | 8.98 ± 0.85 | -0.13 ± 0.78 | 0.90 ± 0.08 |
| KM | 200 | clay | 0.62 ± 0.04 | 8.07 ± 0.46 | 0.35 ± 0.52 | 0.99 ± 0.05 | 0.57 ± 0.07 | 8.78 ± 0.89 | -0.19 ± 0.66 | 0.92 ± 0.09 |
| Random | 250 | clay | 0.63 ± 0.05 | 8.10 ± 0.70 | 0.16 ± 0.67 | 0.99 ± 0.08 | 0.59 ± 0.08 | 8.61 ± 0.98 | -0.26 ± 0.66 | 0.94 ± 0.10 |
| KS | 250 | clay | 0.61* | 8.27* | 1.59* | 0.97* | 0.53* | 9.10* | -0.85* | 0.88* |
| cLHS | 250 | clay | 0.62 ± 0.07 | 8.29 ± 1.21 | 0.03 ± 0.47 | 0.98 ± 0.11 | 0.59 ± 0.06 | 8.50 ± 0.77 | -0.27 ± 0.65 | 0.95 ± 0.08 |
| KM | 250 | clay | 0.64 ± 0.03 | 7.84 ± 0.34 | 0.31 ± 0.50 | 1.02 ± 0.04 | 0.58 ± 0.07 | 8.56 ± 0.81 | -0.25 ± 0.69 | 0.94 ± 0.09 |
| Random | 300 | clay | 0.63 ± 0.05 | 8.06 ± 0.80 | 0.12 ± 0.54 | 1.00 ± 0.09 | 0.60 ± 0.06 | 8.53 ± 0.88 | -0.18 ± 0.73 | 0.95 ± 0.09 |
| KS | 300 | clay | 0.63* | 8.17* | 1.60* | 0.98* | 0.51* | 9.31* | -0.72* | 0.86* |
| cLHS | 300 | clay | 0.63 ± 0.07 | 8.11 ± 1.47 | 0.18 ± 0.45 | 1.00 ± 0.10 | 0.58 ± 0.07 | 8.69 ± 1.00 | 0.04 ± 0.61 | 0.93 ± 0.10 |
| KM | 300 | clay | 0.65 ± 0.06 | 7.81 ± 1.09 | 0.23 ± 0.41 | 1.04 ± 0.08 | 0.59 ± 0.07 | 8.51 ± 0.79 | -0.26 ± 0.55 | 0.95 ± 0.09 |
| Random | 400 | clay | 0.65 ± 0.07 | 7.93 ± 1.43 | 0.14 ± 0.42 | 1.03 ± 0.11 | 0.61 ± 0.07 | 8.31 ± 0.82 | -0.18 ± 0.45 | 0.97 ± 0.09 |
| KS | 400 | clay | 0.65* | 7.91* | 1.33* | 1.01* | 0.51* | 9.20* | -1.40* | 0.87* |
| cLHS | 400 | clay | 0.66 ± 0.03 | 7.70 ± 0.45 | 0.10 ± 0.38 | 1.04 ± 0.05 | 0.61 ± 0.08 | 8.36 ± 0.93 | -0.18 ± 0.54 | 0.97 ± 0.10 |
| KM | 400 | clay | 0.67 ± 0.02 | 7.50 ± 0.30 | 0.30 ± 0.38 | 1.07 ± 0.04 | 0.60 ± 0.06 | 8.37 ± 0.63 | -0.29 ± 0.51 | 0.96 ± 0.07 |
| Random | 500 | clay | 0.67 ± 0.03 | 7.60 ± 0.49 | 0.08 ± 0.38 | 1.06 ± 0.06 | 0.61 ± 0.07 | 8.28 ± 0.86 | -0.35 ± 0.40 | 0.98 ± 0.09 |
| KS | 500 | clay | 0.66* | 7.81* | 1.56* | 1.02* | 0.61* | 8.19* | 0.67* | 0.98* |
| cLHS | 500 | clay | 0.68 ± 0.03 | 7.46 ± 0.39 | 0.11 ± 0.32 | 1.08 ± 0.05 | 0.63 ± 0.07 | 8.03 ± 0.80 | -0.31 ± 0.45 | 1.01 ± 0.10 |
| KM | 500 | clay | 0.67 ± 0.03 | 7.43 ± 0.34 | 0.23 ± 0.30 | 1.08 ± 0.04 | 0.61 ± 0.05 | 8.19 ± 0.63 | -0.23 ± 0.45 | 0.98 ± 0.08 |
| Random | 1000 | clay | 0.70 ± 0.02 | 7.14 ± 0.29 | 0.13 ± 0.20 | 1.12 ± 0.04 | 0.64 ± 0.04 | 7.87 ± 0.54 | -0.25 ± 0.32 | 1.02 ± 0.07 |
| KS | 1000 | clay | 0.70* | 7.24* | 0.77* | 1.10* | 0.63* | 7.91* | -0.26* | 1.01* |
| cLHS | 1000 | clay | 0.70 ± 0.01 | 7.09 ± 0.15 | 0.13 ± 0.20 | 1.13 ± 0.02 | 0.66 ± 0.04 | 7.66 ± 0.47 | -0.24 ± 0.31 | 1.05 ± 0.06 |
| KM | 1000 | clay | 0.70 ± 0.03 | 7.17 ± 0.34 | 0.21 ± 0.19 | 1.12 ± 0.05 | 0.64 ± 0.04 | 7.92 ± 0.54 | -0.19 ± 0.30 | 1.02 ± 0.07 |
| Random | 1500 | clay | 0.72 ± 0.01 | 6.91 ± 0.17 | 0.13 ± 0.14 | 1.16 ± 0.03 | 0.67 ± 0.04 | 7.60 ± 0.48 | -0.17 ± 0.23 | 1.06 ± 0.06 |
| KS | 1500 | clay | 0.71* | 7.09* | 0.78* | 1.13* | 0.63* | 7.96* | 0.43* | 1.01* |
| cLHS | 1500 | clay | 0.72 ± 0.01 | 6.89 ± 0.12 | 0.15 ± 0.17 | 1.16 ± 0.02 | 0.68 ± 0.03 | 7.49 ± 0.40 | -0.16 ± 0.29 | 1.07 ± 0.06 |
| KM | 1500 | clay | 0.72 ± 0.01 | 6.86 ± 0.10 | 0.17 ± 0.15 | 1.17 ± 0.02 | 0.65 ± 0.03 | 7.80 ± 0.45 | -0.09 ± 0.26 | 1.03 ± 0.06 |
| Random | 2000 | clay | 0.73 ± 0.01 | 6.80 ± 0.10 | 0.15 ± 0.11 | 1.18 ± 0.02 | 0.67 ± 0.03 | 7.53 ± 0.36 | -0.18 ± 0.21 | 1.06 ± 0.05 |
| KS | 2000 | clay | 0.72* | 6.91* | 0.67* | 1.16* | 0.73* | 6.72* | -0.18* | 1.19* |
| cLHS | 2000 | clay | 0.73 ± 0.01 | 6.81 ± 0.08 | 0.12 ± 0.11 | 1.17 ± 0.01 | 0.68 ± 0.03 | 7.40 ± 0.35 | -0.14 ± 0.21 | 1.08 ± 0.05 |
| KM | 2000 | clay | 0.73 ± 0.01 | 6.82 ± 0.09 | 0.15 ± 0.15 | 1.17 ± 0.02 | 0.65 ± 0.04 | 7.86 ± 0.60 | -0.02 ± 0.24 | 1.02 ± 0.08 |
| Random | 3000 | clay | 0.73 ± 0.00 | 6.70 ± 0.06 | 0.14 ± 0.08 | 1.19 ± 0.01 | 0.70 ± 0.02 | 7.27 ± 0.31 | -0.08 ± 0.19 | 1.10 ± 0.05 |
| KS | 3000 | clay | 0.73* | 6.76* | 0.60* | 1.18* | 0.68* | 7.33* | -0.15* | 1.09* |
| cLHS | 3000 | clay | 0.73 ± 0.00 | 6.73 ± 0.06 | 0.20 ± 0.08 | 1.19 ± 0.01 | 0.70 ± 0.03 | 7.23 ± 0.34 | -0.10 ± 0.19 | 1.11 ± 0.05 |
| KM | 3000 | clay | 0.72 ± 0.01 | 6.84 ± 0.19 | 0.13 ± 0.18 | 1.17 ± 0.03 | 0.58 ± 0.11 | 9.07 ± 1.71 | 0.06 ± 0.27 | 0.91 ± 0.16 |
| Random | 50 | sand | 0.23 ± 0.07 | 24.84 ± 3.46 | -0.01 ± 3.41 | 0.98 ± 0.10 | 0.19 ± 0.08 | 25.89 ± 2.43 | 0.62 ± 4.06 | 0.93 ± 0.08 |
| KS | 50 | sand | 0.26* | 23.35* | 4.78* | 1.03* | 0.18* | 23.87* | 3.55* | 1.01* |
| cLHS | 50 | sand | 0.26 ± 0.07 | 23.28 ± 1.26 | 0.52 ± 2.93 | 1.03 ± 0.06 | 0.20 ± 0.08 | 25.64 ± 2.55 | 0.47 ± 4.24 | 0.94 ± 0.09 |
| KM | 50 | sand | 0.25 ± 0.07 | 23.32 ± 1.50 | 0.55 ± 3.46 | 1.03 ± 0.06 | 0.20 ± 0.08 | 25.24 ± 2.02 | 1.12 ± 4.87 | 0.96 ± 0.07 |
| Random | 100 | sand | 0.33 ± 0.06 | 22.39 ± 3.52 | 0.15 ± 2.10 | 1.09 ± 0.10 | 0.27 ± 0.08 | 23.67 ± 1.80 | 0.89 ± 3.11 | 1.02 ± 0.08 |
| KS | 100 | sand | 0.36* | 22.04* | -2.20* | 1.09* | 0.30* | 21.93* | -0.86* | 1.09* |
| cLHS | 100 | sand | 0.33 ± 0.05 | 21.87 ± 2.26 | 0.76 ± 1.64 | 1.11 ± 0.08 | 0.29 ± 0.06 | 22.87 ± 1.42 | 0.97 ± 2.28 | 1.05 ± 0.06 |
| KM | 100 | sand | 0.32 ± 0.06 | 22.07 ± 2.24 | -0.14 ± 2.44 | 1.09 ± 0.08 | 0.29 ± 0.06 | 23.13 ± 1.54 | 0.56 ± 3.32 | 1.04 ± 0.07 |
| Random | 150 | sand | 0.35 ± 0.07 | 22.41 ± 5.69 | -0.06 ± 1.90 | 1.10 ± 0.14 | 0.33 ± 0.06 | 22.15 ± 1.13 | 0.21 ± 2.34 | 1.09 ± 0.06 |
| KS | 150 | sand | 0.35* | 21.52* | 1.75* | 1.12* | 0.35* | 21.66* | 4.37* | 1.11* |
| cLHS | 150 | sand | 0.37 ± 0.05 | 21.53 ± 3.87 | -0.03 ± 1.83 | 1.13 ± 0.10 | 0.33 ± 0.05 | 22.21 ± 1.10 | 0.23 ± 2.17 | 1.08 ± 0.05 |
| KM | 150 | sand | 0.38 ± 0.04 | 20.73 ± 0.70 | -0.35 ± 1.55 | 1.16 ± 0.04 | 0.34 ± 0.04 | 21.85 ± 1.06 | -0.16 ± 2.32 | 1.10 ± 0.05 |
| Random | 200 | sand | 0.37 ± 0.05 | 21.41 ± 2.59 | 0.04 ± 1.62 | 1.13 ± 0.09 | 0.36 ± 0.05 | 21.56 ± 1.25 | 0.01 ± 2.26 | 1.12 ± 0.06 |
| KS | 200 | sand | 0.38* | 21.35* | 2.52* | 1.12* | 0.37* | 21.35* | 2.97* | 1.12* |
| cLHS | 200 | sand | 0.35 ± 0.09 | 23.35 ± 8.82 | 0.12 ± 1.73 | 1.09 ± 0.18 | 0.36 ± 0.06 | 21.69 ± 1.38 | 0.08 ± 2.26 | 1.11 ± 0.07 |
| KM | 200 | sand | 0.40 ± 0.03 | 20.35 ± 0.58 | -0.16 ± 1.47 | 1.18 ± 0.03 | 0.37 ± 0.06 | 21.21 ± 1.28 | 0.76 ± 1.87 | 1.14 ± 0.06 |
| Random | 250 | sand | 0.38 ± 0.07 | 21.54 ± 3.68 | 0.10 ± 1.51 | 1.13 ± 0.13 | 0.37 ± 0.04 | 21.30 ± 0.90 | 0.26 ± 1.98 | 1.13 ± 0.05 |
| KS | 250 | sand | 0.41* | 20.68* | 1.77* | 1.16* | 0.34* | 21.29* | 0.24* | 1.13* |
| cLHS | 250 | sand | 0.38 ± 0.06 | 21.45 ± 2.77 | -0.04 ± 1.48 | 1.13 ± 0.11 | 0.38 ± 0.06 | 21.21 ± 1.28 | 0.32 ± 1.89 | 1.14 ± 0.07 |
| KM | 250 | sand | 0.41 ± 0.03 | 20.11 ± 0.53 | -0.19 ± 1.25 | 1.19 ± 0.03 | 0.40 ± 0.05 | 20.62 ± 0.99 | 0.09 ± 1.70 | 1.17 ± 0.06 |
| Random | 300 | sand | 0.37 ± 0.09 | 22.33 ± 4.45 | 0.35 ± 1.39 | 1.11 ± 0.16 | 0.40 ± 0.05 | 20.77 ± 0.98 | 0.45 ± 1.58 | 1.16 ± 0.05 |
| KS | 300 | sand | 0.43* | 20.34* | 0.06* | 1.18* | 0.33* | 21.76* | 2.71* | 1.10* |
| cLHS | 300 | sand | 0.39 ± 0.07 | 21.72 ± 5.28 | 0.18 ± 0.91 | 1.14 ± 0.14 | 0.41 ± 0.05 | 20.54 ± 1.06 | 0.24 ± 1.36 | 1.17 ± 0.06 |
| KM | 300 | sand | 0.42 ± 0.04 | 20.07 ± 1.33 | -0.20 ± 1.27 | 1.20 ± 0.06 | 0.40 ± 0.05 | 20.65 ± 1.15 | 0.47 ± 1.94 | 1.17 ± 0.06 |
| Random | 400 | sand | 0.39 ± 0.09 | 21.86 ± 4.69 | 0.26 ± 1.09 | 1.13 ± 0.16 | 0.41 ± 0.05 | 20.61 ± 1.20 | 0.13 ± 1.45 | 1.17 ± 0.07 |
| KS | 400 | sand | 0.46* | 19.41* | -1.27* | 1.24* | 0.38* | 20.54* | 1.23* | 1.17* |
| cLHS | 400 | sand | 0.43 ± 0.04 | 20.04 ± 1.10 | 0.20 ± 1.02 | 1.20 ± 0.06 | 0.41 ± 0.05 | 20.58 ± 1.18 | 0.52 ± 1.27 | 1.17 ± 0.07 |
| KM | 400 | sand | 0.44 ± 0.02 | 19.57 ± 0.44 | -0.35 ± 0.98 | 1.23 ± 0.03 | 0.41 ± 0.04 | 20.30 ± 0.87 | 0.17 ± 1.21 | 1.18 ± 0.05 |
| Random | 500 | sand | 0.41 ± 0.10 | 21.41 ± 4.58 | 0.47 ± 1.04 | 1.16 ± 0.17 | 0.41 ± 0.06 | 20.64 ± 1.14 | 0.10 ± 1.28 | 1.17 ± 0.06 |
| KS | 500 | sand | 0.45* | 20.29* | -1.36* | 1.18* | 0.26* | 22.57* | -2.01* | 1.06* |
| cLHS | 500 | sand | 0.44 ± 0.09 | 20.61 ± 5.02 | 0.15 ± 1.02 | 1.20 ± 0.16 | 0.41 ± 0.04 | 20.68 ± 0.92 | 0.27 ± 1.56 | 1.16 ± 0.05 |
| KM | 500 | sand | 0.46 ± 0.02 | 19.18 ± 0.43 | -0.28 ± 0.92 | 1.25 ± 0.03 | 0.41 ± 0.05 | 20.40 ± 1.08 | 0.19 ± 1.14 | 1.18 ± 0.06 |
| Random | 1000 | sand | 0.50 ± 0.02 | 18.59 ± 0.42 | 0.13 ± 0.62 | 1.29 ± 0.03 | 0.46 ± 0.04 | 19.70 ± 0.80 | 0.20 ± 0.87 | 1.22 ± 0.05 |
| KS | 1000 | sand | 0.49* | 18.85* | -0.56* | 1.27* | 0.35* | 21.21* | 1.58* | 1.13* |
| cLHS | 1000 | sand | 0.50 ± 0.01 | 18.50 ± 0.27 | 0.08 ± 0.52 | 1.30 ± 0.02 | 0.46 ± 0.04 | 19.49 ± 0.74 | 0.28 ± 0.80 | 1.23 ± 0.05 |
| KM | 1000 | sand | 0.50 ± 0.03 | 18.50 ± 0.78 | -0.26 ± 0.52 | 1.30 ± 0.05 | 0.46 ± 0.04 | 19.62 ± 0.84 | 0.11 ± 0.71 | 1.23 ± 0.05 |
| Random | 1500 | sand | 0.51 ± 0.01 | 18.21 ± 0.26 | 0.09 ± 0.43 | 1.32 ± 0.02 | 0.49 ± 0.04 | 19.10 ± 0.75 | 0.06 ± 0.55 | 1.26 ± 0.05 |
| KS | 1500 | sand | 0.51* | 18.42* | -0.19* | 1.30* | 0.41* | 20.51* | -0.64* | 1.17* |
| cLHS | 1500 | sand | 0.52 ± 0.01 | 18.13 ± 0.17 | -0.10 ± 0.41 | 1.32 ± 0.01 | 0.49 ± 0.03 | 19.11 ± 0.63 | -0.09 ± 0.60 | 1.26 ± 0.04 |
| KM | 1500 | sand | 0.51 ± 0.01 | 18.24 ± 0.33 | -0.03 ± 0.40 | 1.32 ± 0.02 | 0.47 ± 0.04 | 19.59 ± 0.83 | 0.20 ± 0.83 | 1.23 ± 0.05 |
| Random | 2000 | sand | 0.52 ± 0.01 | 18.03 ± 0.17 | 0.01 ± 0.31 | 1.33 ± 0.01 | 0.50 ± 0.03 | 18.71 ± 0.61 | 0.15 ± 0.65 | 1.28 ± 0.04 |
| KS | 2000 | sand | 0.52* | 18.16* | -0.20* | 1.32* | 0.41* | 20.06* | -0.89* | 1.20* |
| cLHS | 2000 | sand | 0.52 ± 0.01 | 18.01 ± 0.16 | -0.04 ± 0.29 | 1.33 ± 0.01 | 0.51 ± 0.03 | 18.70 ± 0.70 | -0.02 ± 0.60 | 1.29 ± 0.05 |
| KM | 2000 | sand | 0.51 ± 0.01 | 18.20 ± 0.29 | 0.04 ± 0.47 | 1.32 ± 0.02 | 0.47 ± 0.04 | 19.67 ± 0.87 | 0.25 ± 0.66 | 1.22 ± 0.05 |
| Random | 3000 | sand | 0.53 ± 0.00 | 17.86 ± 0.10 | -0.04 ± 0.23 | 1.34 ± 0.01 | 0.52 ± 0.03 | 18.44 ± 0.61 | 0.19 ± 0.46 | 1.30 ± 0.04 |
| KS | 3000 | sand | 0.53* | 17.87* | -0.69* | 1.34* | 0.54* | 17.79* | 0.19* | 1.35* |
| cLHS | 3000 | sand | 0.53 ± 0.00 | 17.85 ± 0.07 | -0.20 ± 0.22 | 1.34 ± 0.00 | 0.52 ± 0.02 | 18.38 ± 0.42 | 0.06 ± 0.57 | 1.31 ± 0.03 |
| KM | 3000 | sand | 0.52 ± 0.02 | 18.19 ± 0.49 | 0.05 ± 0.73 | 1.32 ± 0.03 | 0.41 ± 0.08 | 22.07 ± 2.71 | 0.75 ± 0.90 | 1.10 ± 0.13 |
| Random | 50 | pH.in.CaCl2 | 0.49 ± 0.09 | 1.12 ± 0.43 | 0.02 ± 0.14 | 1.10 ± 0.19 | 0.36 ± 0.12 | 1.18 ± 0.15 | 0.06 ± 0.22 | 1.00 ± 0.13 |
| KS | 50 | pH.in.CaCl2 | 0.56* | 0.95* | -0.19* | 1.22* | 0.37* | 1.08* | -0.27* | 1.07* |
| cLHS | 50 | pH.in.CaCl2 | 0.52 ± 0.10 | 1.06 ± 0.32 | 0.04 ± 0.11 | 1.15 ± 0.21 | 0.41 ± 0.10 | 1.09 ± 0.14 | 0.10 ± 0.19 | 1.07 ± 0.12 |
| KM | 50 | pH.in.CaCl2 | 0.53 ± 0.08 | 0.93 ± 0.08 | 0.07 ± 0.10 | 1.25 ± 0.10 | 0.40 ± 0.11 | 1.08 ± 0.13 | 0.08 ± 0.15 | 1.09 ± 0.13 |
| Random | 100 | pH.in.CaCl2 | 0.56 ± 0.09 | 0.98 ± 0.22 | 0.00 ± 0.09 | 1.23 ± 0.20 | 0.55 ± 0.08 | 0.93 ± 0.10 | 0.06 ± 0.10 | 1.25 ± 0.13 |
| KS | 100 | pH.in.CaCl2 | 0.54* | 0.96* | -0.08* | 1.20* | 0.21* | 1.32* | -0.04* | 0.88* |
| cLHS | 100 | pH.in.CaCl2 | 0.58 ± 0.08 | 0.93 ± 0.18 | 0.03 ± 0.08 | 1.28 ± 0.19 | 0.57 ± 0.05 | 0.90 ± 0.06 | 0.05 ± 0.11 | 1.29 ± 0.09 |
| KM | 100 | pH.in.CaCl2 | 0.62 ± 0.05 | 0.84 ± 0.12 | 0.03 ± 0.09 | 1.39 ± 0.11 | 0.54 ± 0.08 | 0.92 ± 0.09 | 0.03 ± 0.12 | 1.27 ± 0.12 |
| Random | 150 | pH.in.CaCl2 | 0.60 ± 0.12 | 0.97 ± 0.37 | 0.01 ± 0.07 | 1.29 ± 0.28 | 0.62 ± 0.07 | 0.85 ± 0.10 | 0.06 ± 0.09 | 1.38 ± 0.15 |
| KS | 150 | pH.in.CaCl2 | 0.70* | 0.74* | -0.15* | 1.56* | 0.56* | 0.93* | -0.30* | 1.24* |
| cLHS | 150 | pH.in.CaCl2 | 0.61 ± 0.11 | 0.93 ± 0.31 | 0.01 ± 0.06 | 1.32 ± 0.26 | 0.63 ± 0.07 | 0.83 ± 0.07 | 0.03 ± 0.09 | 1.40 ± 0.11 |
| KM | 150 | pH.in.CaCl2 | 0.65 ± 0.05 | 0.80 ± 0.09 | 0.04 ± 0.07 | 1.46 ± 0.12 | 0.62 ± 0.07 | 0.83 ± 0.09 | 0.04 ± 0.10 | 1.41 ± 0.14 |
| Random | 200 | pH.in.CaCl2 | 0.63 ± 0.13 | 0.91 ± 0.33 | 0.01 ± 0.05 | 1.38 ± 0.30 | 0.65 ± 0.06 | 0.80 ± 0.08 | 0.04 ± 0.07 | 1.45 ± 0.13 |
| KS | 200 | pH.in.CaCl2 | 0.72* | 0.72* | -0.12* | 1.60* | 0.53* | 0.94* | -0.24* | 1.23* |
| cLHS | 200 | pH.in.CaCl2 | 0.65 ± 0.09 | 0.83 ± 0.17 | 0.02 ± 0.05 | 1.43 ± 0.22 | 0.65 ± 0.06 | 0.80 ± 0.07 | 0.04 ± 0.07 | 1.47 ± 0.13 |
| KM | 200 | pH.in.CaCl2 | 0.70 ± 0.04 | 0.74 ± 0.05 | 0.03 ± 0.05 | 1.57 ± 0.10 | 0.66 ± 0.07 | 0.79 ± 0.08 | 0.05 ± 0.07 | 1.49 ± 0.14 |
| Random | 250 | pH.in.CaCl2 | 0.67 ± 0.09 | 0.82 ± 0.18 | 0.01 ± 0.05 | 1.48 ± 0.26 | 0.69 ± 0.06 | 0.76 ± 0.07 | 0.05 ± 0.06 | 1.54 ± 0.14 |
| KS | 250 | pH.in.CaCl2 | 0.72* | 0.74* | -0.09* | 1.57* | 0.63* | 0.81* | -0.02* | 1.43* |
| cLHS | 250 | pH.in.CaCl2 | 0.67 ± 0.11 | 0.84 ± 0.27 | 0.02 ± 0.05 | 1.47 ± 0.29 | 0.69 ± 0.05 | 0.76 ± 0.07 | 0.04 ± 0.05 | 1.54 ± 0.14 |
| KM | 250 | pH.in.CaCl2 | 0.71 ± 0.05 | 0.72 ± 0.06 | 0.03 ± 0.03 | 1.62 ± 0.14 | 0.70 ± 0.06 | 0.73 ± 0.07 | 0.05 ± 0.04 | 1.59 ± 0.15 |
| Random | 300 | pH.in.CaCl2 | 0.68 ± 0.09 | 0.79 ± 0.17 | 0.02 ± 0.04 | 1.52 ± 0.25 | 0.71 ± 0.05 | 0.73 ± 0.07 | 0.04 ± 0.04 | 1.61 ± 0.13 |
| KS | 300 | pH.in.CaCl2 | 0.70* | 0.75* | -0.02* | 1.55* | 0.66* | 0.78* | -0.07* | 1.48* |
| cLHS | 300 | pH.in.CaCl2 | 0.69 ± 0.11 | 0.79 ± 0.27 | 0.02 ± 0.04 | 1.55 ± 0.28 | 0.70 ± 0.06 | 0.73 ± 0.07 | 0.05 ± 0.06 | 1.60 ± 0.15 |
| KM | 300 | pH.in.CaCl2 | 0.71 ± 0.07 | 0.73 ± 0.14 | 0.04 ± 0.04 | 1.63 ± 0.22 | 0.69 ± 0.07 | 0.74 ± 0.08 | 0.05 ± 0.05 | 1.57 ± 0.17 |
| Random | 400 | pH.in.CaCl2 | 0.72 ± 0.08 | 0.73 ± 0.16 | 0.02 ± 0.04 | 1.64 ± 0.24 | 0.72 ± 0.05 | 0.71 ± 0.07 | 0.04 ± 0.05 | 1.64 ± 0.16 |
| KS | 400 | pH.in.CaCl2 | 0.76* | 0.67* | -0.03* | 1.72* | 0.73* | 0.69* | -0.02* | 1.67* |
| cLHS | 400 | pH.in.CaCl2 | 0.72 ± 0.10 | 0.74 ± 0.27 | 0.02 ± 0.03 | 1.65 ± 0.28 | 0.73 ± 0.06 | 0.69 ± 0.08 | 0.04 ± 0.04 | 1.69 ± 0.17 |
| KM | 400 | pH.in.CaCl2 | 0.75 ± 0.04 | 0.67 ± 0.05 | 0.02 ± 0.03 | 1.75 ± 0.13 | 0.72 ± 0.06 | 0.70 ± 0.08 | 0.03 ± 0.04 | 1.66 ± 0.18 |
| Random | 500 | pH.in.CaCl2 | 0.74 ± 0.07 | 0.70 ± 0.12 | 0.02 ± 0.04 | 1.69 ± 0.22 | 0.73 ± 0.04 | 0.70 ± 0.06 | 0.04 ± 0.04 | 1.68 ± 0.15 |
| KS | 500 | pH.in.CaCl2 | 0.76* | 0.67* | -0.03* | 1.74* | 0.71* | 0.72* | -0.04* | 1.61* |
| cLHS | 500 | pH.in.CaCl2 | 0.75 ± 0.07 | 0.68 ± 0.12 | 0.01 ± 0.03 | 1.73 ± 0.21 | 0.74 ± 0.05 | 0.68 ± 0.07 | 0.03 ± 0.04 | 1.72 ± 0.17 |
| KM | 500 | pH.in.CaCl2 | 0.76 ± 0.04 | 0.65 ± 0.05 | 0.02 ± 0.03 | 1.79 ± 0.13 | 0.73 ± 0.06 | 0.69 ± 0.08 | 0.04 ± 0.04 | 1.70 ± 0.18 |
| Random | 1000 | pH.in.CaCl2 | 0.78 ± 0.05 | 0.62 ± 0.10 | 0.02 ± 0.02 | 1.88 ± 0.17 | 0.77 ± 0.04 | 0.64 ± 0.06 | 0.04 ± 0.03 | 1.82 ± 0.16 |
| KS | 1000 | pH.in.CaCl2 | 0.79* | 0.63* | 0.00* | 1.85* | 0.83* | 0.55* | -0.01* | 2.12* |
| cLHS | 1000 | pH.in.CaCl2 | 0.78 ± 0.04 | 0.62 ± 0.05 | 0.02 ± 0.02 | 1.89 ± 0.13 | 0.78 ± 0.04 | 0.63 ± 0.05 | 0.04 ± 0.03 | 1.86 ± 0.17 |
| KM | 1000 | pH.in.CaCl2 | 0.79 ± 0.03 | 0.61 ± 0.04 | 0.02 ± 0.02 | 1.89 ± 0.10 | 0.77 ± 0.04 | 0.64 ± 0.06 | 0.04 ± 0.02 | 1.83 ± 0.17 |
| Random | 1500 | pH.in.CaCl2 | 0.80 ± 0.02 | 0.59 ± 0.02 | 0.02 ± 0.01 | 1.97 ± 0.07 | 0.79 ± 0.03 | 0.61 ± 0.05 | 0.04 ± 0.02 | 1.92 ± 0.16 |
| KS | 1500 | pH.in.CaCl2 | 0.80* | 0.61* | 0.01* | 1.90* | 0.76* | 0.65* | -0.03* | 1.79* |
| cLHS | 1500 | pH.in.CaCl2 | 0.81 ± 0.01 | 0.58 ± 0.01 | 0.02 ± 0.01 | 1.98 ± 0.03 | 0.80 ± 0.03 | 0.59 ± 0.04 | 0.04 ± 0.02 | 1.96 ± 0.14 |
| KM | 1500 | pH.in.CaCl2 | 0.80 ± 0.02 | 0.59 ± 0.02 | 0.02 ± 0.01 | 1.97 ± 0.06 | 0.79 ± 0.02 | 0.62 ± 0.04 | 0.03 ± 0.03 | 1.88 ± 0.12 |
| Random | 2000 | pH.in.CaCl2 | 0.81 ± 0.01 | 0.58 ± 0.01 | 0.02 ± 0.01 | 2.00 ± 0.03 | 0.81 ± 0.02 | 0.57 ± 0.03 | 0.04 ± 0.02 | 2.02 ± 0.12 |
| KS | 2000 | pH.in.CaCl2 | 0.80* | 0.60* | 0.02* | 1.94* | 0.80* | 0.60* | 0.04* | 1.94* |
| cLHS | 2000 | pH.in.CaCl2 | 0.81 ± 0.01 | 0.58 ± 0.01 | 0.02 ± 0.01 | 2.00 ± 0.03 | 0.81 ± 0.03 | 0.58 ± 0.04 | 0.04 ± 0.02 | 2.02 ± 0.15 |
| KM | 2000 | pH.in.CaCl2 | 0.81 ± 0.01 | 0.58 ± 0.01 | 0.02 ± 0.01 | 1.99 ± 0.04 | 0.79 ± 0.04 | 0.61 ± 0.06 | 0.04 ± 0.02 | 1.90 ± 0.17 |
| Random | 3000 | pH.in.CaCl2 | 0.81 ± 0.00 | 0.57 ± 0.00 | 0.02 ± 0.01 | 2.03 ± 0.02 | 0.83 ± 0.02 | 0.55 ± 0.04 | 0.03 ± 0.02 | 2.10 ± 0.15 |
| KS | 3000 | pH.in.CaCl2 | 0.80* | 0.59* | 0.02* | 1.98* | 0.84* | 0.53* | -0.01* | 2.18* |
| cLHS | 3000 | pH.in.CaCl2 | 0.81 ± 0.00 | 0.58 ± 0.01 | 0.02 ± 0.01 | 2.01 ± 0.02 | 0.83 ± 0.02 | 0.55 ± 0.04 | 0.03 ± 0.02 | 2.10 ± 0.14 |
| KM | 3000 | pH.in.CaCl2 | 0.81 ± 0.01 | 0.58 ± 0.02 | 0.02 ± 0.01 | 1.99 ± 0.06 | 0.73 ± 0.10 | 0.71 ± 0.15 | 0.03 ± 0.02 | 1.69 ± 0.31 |
| Random | 50 | Organic Carbon | 0.29 ± 0.07 | 17.50 ± 2.22 | -0.67 ± 2.03 | 0.43 ± 0.05 | 0.24 ± 0.11 | 18.54 ± 3.25 | -2.28 ± 2.90 | 0.41 ± 0.05 |
| KS | 50 | Organic Carbon | 0.35* | 16.39* | 5.96* | 0.45* | 0.19* | 17.61* | 5.10* | 0.42* |
| cLHS | 50 | Organic Carbon | 0.29 ± 0.07 | 17.34 ± 4.43 | -0.46 ± 1.84 | 0.44 ± 0.06 | 0.24 ± 0.08 | 18.34 ± 2.44 | -1.81 ± 2.77 | 0.41 ± 0.05 |
| KM | 50 | Organic Carbon | 0.29 ± 0.08 | 16.38 ± 1.37 | -0.21 ± 2.04 | 0.45 ± 0.03 | 0.22 ± 0.08 | 18.00 ± 2.01 | -1.68 ± 2.65 | 0.42 ± 0.04 |
| Random | 100 | Organic Carbon | 0.39 ± 0.06 | 15.64 ± 1.56 | 0.21 ± 1.39 | 0.48 ± 0.04 | 0.31 ± 0.09 | 16.90 ± 2.19 | -1.07 ± 1.98 | 0.44 ± 0.05 |
| KS | 100 | Organic Carbon | 0.32* | 17.01* | 6.89* | 0.44* | 0.26* | 17.54* | 6.70* | 0.42* |
| cLHS | 100 | Organic Carbon | 0.40 ± 0.08 | 15.69 ± 3.02 | -0.36 ± 1.53 | 0.48 ± 0.06 | 0.29 ± 0.09 | 17.13 ± 1.60 | -2.00 ± 2.22 | 0.44 ± 0.04 |
| KM | 100 | Organic Carbon | 0.40 ± 0.07 | 15.03 ± 1.42 | 0.43 ± 1.53 | 0.50 ± 0.04 | 0.28 ± 0.09 | 17.35 ± 2.24 | -0.94 ± 2.06 | 0.43 ± 0.05 |
| Random | 150 | Organic Carbon | 0.44 ± 0.07 | 14.86 ± 2.66 | 0.30 ± 1.09 | 0.51 ± 0.05 | 0.35 ± 0.09 | 16.23 ± 2.06 | -0.95 ± 1.47 | 0.46 ± 0.05 |
| KS | 150 | Organic Carbon | 0.44* | 14.57* | 3.59* | 0.51* | 0.10* | 19.34* | -0.92* | 0.38* |
| cLHS | 150 | Organic Carbon | 0.43 ± 0.09 | 15.28 ± 3.06 | 0.05 ± 1.13 | 0.50 ± 0.07 | 0.36 ± 0.08 | 15.73 ± 1.50 | -1.02 ± 1.48 | 0.47 ± 0.04 |
| KM | 150 | Organic Carbon | 0.46 ± 0.07 | 14.06 ± 0.84 | 0.12 ± 1.10 | 0.53 ± 0.03 | 0.36 ± 0.10 | 15.72 ± 1.75 | -1.13 ± 1.40 | 0.48 ± 0.05 |
| Random | 200 | Organic Carbon | 0.47 ± 0.07 | 14.32 ± 2.11 | 0.21 ± 1.09 | 0.52 ± 0.05 | 0.37 ± 0.08 | 16.02 ± 2.02 | -1.19 ± 1.39 | 0.47 ± 0.05 |
| KS | 200 | Organic Carbon | 0.45* | 14.60* | 4.25* | 0.51* | 0.14* | 19.81* | 3.18* | 0.37* |
| cLHS | 200 | Organic Carbon | 0.47 ± 0.04 | 14.06 ± 0.76 | -0.02 ± 0.94 | 0.53 ± 0.03 | 0.38 ± 0.08 | 15.69 ± 1.77 | -0.97 ± 1.32 | 0.48 ± 0.05 |
| KM | 200 | Organic Carbon | 0.49 ± 0.06 | 13.54 ± 1.12 | 0.13 ± 0.98 | 0.55 ± 0.04 | 0.34 ± 0.10 | 15.90 ± 1.55 | -1.42 ± 1.31 | 0.47 ± 0.05 |
| Random | 250 | Organic Carbon | 0.48 ± 0.06 | 13.98 ± 2.22 | 0.06 ± 0.91 | 0.54 ± 0.05 | 0.39 ± 0.09 | 15.59 ± 1.68 | -1.23 ± 1.53 | 0.48 ± 0.05 |
| KS | 250 | Organic Carbon | 0.48* | 14.10* | 3.56* | 0.52* | 0.33* | 15.39* | -0.03* | 0.48* |
| cLHS | 250 | Organic Carbon | 0.48 ± 0.07 | 14.15 ± 2.33 | 0.01 ± 0.94 | 0.53 ± 0.06 | 0.38 ± 0.09 | 15.58 ± 1.53 | -1.01 ± 1.32 | 0.48 ± 0.05 |
| KM | 250 | Organic Carbon | 0.52 ± 0.04 | 13.25 ± 0.75 | 0.26 ± 0.76 | 0.56 ± 0.03 | 0.38 ± 0.08 | 15.55 ± 1.33 | -0.79 ± 1.36 | 0.48 ± 0.04 |
| Random | 300 | Organic Carbon | 0.49 ± 0.08 | 14.09 ± 2.80 | 0.05 ± 0.79 | 0.54 ± 0.06 | 0.41 ± 0.08 | 14.92 ± 1.18 | -1.39 ± 1.03 | 0.50 ± 0.04 |
| KS | 300 | Organic Carbon | 0.47* | 14.02* | 2.61* | 0.53* | 0.26* | 16.34* | -1.67* | 0.45* |
| cLHS | 300 | Organic Carbon | 0.49 ± 0.07 | 13.78 ± 2.00 | -0.10 ± 0.65 | 0.54 ± 0.05 | 0.40 ± 0.08 | 15.30 ± 1.39 | -1.17 ± 1.14 | 0.49 ± 0.04 |
| KM | 300 | Organic Carbon | 0.50 ± 0.07 | 13.52 ± 1.48 | 0.14 ± 0.78 | 0.55 ± 0.05 | 0.39 ± 0.08 | 15.21 ± 1.19 | -1.09 ± 1.10 | 0.49 ± 0.04 |
| Random | 400 | Organic Carbon | 0.50 ± 0.08 | 13.77 ± 2.15 | -0.04 ± 0.64 | 0.55 ± 0.06 | 0.43 ± 0.06 | 14.65 ± 0.97 | -1.45 ± 0.83 | 0.51 ± 0.03 |
| KS | 400 | Organic Carbon | 0.48* | 13.81* | 2.30* | 0.54* | 0.22* | 16.84* | -0.77* | 0.44* |
| cLHS | 400 | Organic Carbon | 0.51 ± 0.05 | 13.33 ± 1.14 | -0.12 ± 0.61 | 0.56 ± 0.04 | 0.44 ± 0.06 | 14.49 ± 1.00 | -1.50 ± 0.87 | 0.51 ± 0.04 |
| KM | 400 | Organic Carbon | 0.53 ± 0.02 | 12.92 ± 0.31 | 0.18 ± 0.67 | 0.57 ± 0.01 | 0.44 ± 0.07 | 14.52 ± 1.07 | -1.25 ± 0.98 | 0.51 ± 0.04 |
| Random | 500 | Organic Carbon | 0.51 ± 0.09 | 13.68 ± 2.63 | -0.13 ± 0.56 | 0.55 ± 0.06 | 0.45 ± 0.06 | 14.36 ± 0.97 | -1.53 ± 0.86 | 0.52 ± 0.03 |
| KS | 500 | Organic Carbon | 0.49* | 13.79* | 3.06* | 0.54* | 0.31* | 18.17* | 5.12* | 0.41* |
| cLHS | 500 | Organic Carbon | 0.52 ± 0.06 | 13.23 ± 1.45 | -0.23 ± 0.59 | 0.56 ± 0.04 | 0.46 ± 0.07 | 14.30 ± 1.21 | -1.38 ± 0.92 | 0.52 ± 0.04 |
| KM | 500 | Organic Carbon | 0.54 ± 0.02 | 12.79 ± 0.33 | 0.08 ± 0.53 | 0.58 ± 0.01 | 0.46 ± 0.06 | 14.22 ± 1.01 | -1.34 ± 0.92 | 0.52 ± 0.04 |
| Random | 1000 | Organic Carbon | 0.56 ± 0.02 | 12.47 ± 0.33 | -0.32 ± 0.37 | 0.59 ± 0.01 | 0.51 ± 0.05 | 13.46 ± 0.86 | -1.42 ± 0.57 | 0.55 ± 0.04 |
| KS | 1000 | Organic Carbon | 0.54* | 12.90* | 1.44* | 0.57* | 0.46* | 13.88* | 1.56* | 0.53* |
| cLHS | 1000 | Organic Carbon | 0.57 ± 0.01 | 12.32 ± 0.18 | -0.33 ± 0.31 | 0.60 ± 0.01 | 0.50 ± 0.05 | 13.59 ± 0.80 | -1.60 ± 0.41 | 0.55 ± 0.03 |
| KM | 1000 | Organic Carbon | 0.57 ± 0.01 | 12.31 ± 0.17 | -0.13 ± 0.32 | 0.60 ± 0.01 | 0.51 ± 0.05 | 13.44 ± 0.86 | -1.46 ± 0.58 | 0.55 ± 0.03 |
| Random | 1500 | Organic Carbon | 0.58 ± 0.01 | 12.19 ± 0.13 | -0.29 ± 0.27 | 0.61 ± 0.01 | 0.53 ± 0.04 | 13.05 ± 0.65 | -1.49 ± 0.50 | 0.57 ± 0.03 |
| KS | 1500 | Organic Carbon | 0.56* | 12.47* | 0.84* | 0.59* | 0.58* | 12.19* | 0.42* | 0.61* |
| cLHS | 1500 | Organic Carbon | 0.58 ± 0.01 | 12.18 ± 0.11 | -0.25 ± 0.27 | 0.61 ± 0.01 | 0.54 ± 0.05 | 13.03 ± 0.72 | -1.45 ± 0.47 | 0.57 ± 0.03 |
| KM | 1500 | Organic Carbon | 0.58 ± 0.01 | 12.20 ± 0.16 | -0.22 ± 0.36 | 0.61 ± 0.01 | 0.52 ± 0.05 | 13.24 ± 0.91 | -1.49 ± 0.54 | 0.56 ± 0.04 |
| Random | 2000 | Organic Carbon | 0.59 ± 0.01 | 12.06 ± 0.10 | -0.30 ± 0.19 | 0.61 ± 0.00 | 0.54 ± 0.04 | 12.95 ± 0.64 | -1.47 ± 0.47 | 0.57 ± 0.03 |
| KS | 2000 | Organic Carbon | 0.58* | 12.19* | 0.67* | 0.61* | 0.55* | 12.76* | -0.21* | 0.58* |
| cLHS | 2000 | Organic Carbon | 0.59 ± 0.01 | 12.06 ± 0.10 | -0.39 ± 0.19 | 0.61 ± 0.00 | 0.55 ± 0.04 | 12.83 ± 0.62 | -1.61 ± 0.49 | 0.58 ± 0.03 |
| KM | 2000 | Organic Carbon | 0.58 ± 0.01 | 12.21 ± 0.19 | -0.29 ± 0.38 | 0.61 ± 0.01 | 0.53 ± 0.04 | 13.15 ± 0.69 | -1.35 ± 0.47 | 0.56 ± 0.03 |
| Random | 3000 | Organic Carbon | 0.59 ± 0.00 | 11.96 ± 0.07 | -0.34 ± 0.13 | 0.62 ± 0.00 | 0.58 ± 0.03 | 12.30 ± 0.46 | -1.51 ± 0.38 | 0.60 ± 0.02 |
| KS | 3000 | Organic Carbon | 0.60* | 11.91* | 0.48* | 0.62* | 0.67* | 10.75* | -0.30* | 0.69* |
| cLHS | 3000 | Organic Carbon | 0.59 ± 0.00 | 11.98 ± 0.06 | -0.20 ± 0.14 | 0.62 ± 0.00 | 0.58 ± 0.04 | 12.36 ± 0.57 | -1.45 ± 0.36 | 0.60 ± 0.03 |
| KM | 3000 | Organic Carbon | 0.58 ± 0.02 | 12.30 ± 0.45 | -0.37 ± 0.34 | 0.60 ± 0.02 | 0.46 ± 0.09 | 14.77 ± 2.08 | -1.13 ± 0.51 | 0.51 ± 0.07 |
| Random | 50 | Cation Exchange Capacity | 0.31 ± 0.11 | 9.52 ± 1.78 | 0.02 ± 1.02 | 0.50 ± 0.08 | 0.21 ± 0.11 | 9.75 ± 1.20 | -0.70 ± 1.50 | 0.48 ± 0.05 |
| KS | 50 | Cation Exchange Capacity | 0.40* | 8.19* | 2.61* | 0.57* | 0.28* | 8.98* | 2.99* | 0.52* |
| cLHS | 50 | Cation Exchange Capacity | 0.29 ± 0.08 | 9.58 ± 1.94 | -0.32 ± 1.05 | 0.50 ± 0.07 | 0.23 ± 0.08 | 9.23 ± 0.85 | -0.50 ± 1.26 | 0.51 ± 0.04 |
| KM | 50 | Cation Exchange Capacity | 0.28 ± 0.09 | 8.77 ± 0.71 | 0.21 ± 1.11 | 0.53 ± 0.04 | 0.21 ± 0.10 | 9.41 ± 1.09 | -0.60 ± 1.24 | 0.50 ± 0.05 |
| Random | 100 | Cation Exchange Capacity | 0.42 ± 0.09 | 8.26 ± 1.38 | -0.07 ± 0.76 | 0.57 ± 0.07 | 0.35 ± 0.09 | 8.47 ± 1.26 | -0.55 ± 1.04 | 0.56 ± 0.06 |
| KS | 100 | Cation Exchange Capacity | 0.46* | 8.16* | 3.07* | 0.57* | 0.34* | 8.20* | 1.47* | 0.57* |
| cLHS | 100 | Cation Exchange Capacity | 0.43 ± 0.09 | 8.11 ± 1.22 | -0.21 ± 0.68 | 0.58 ± 0.06 | 0.34 ± 0.11 | 8.60 ± 1.23 | -0.52 ± 0.79 | 0.55 ± 0.07 |
| KM | 100 | Cation Exchange Capacity | 0.45 ± 0.09 | 7.53 ± 0.66 | 0.20 ± 0.71 | 0.62 ± 0.05 | 0.37 ± 0.08 | 8.14 ± 0.68 | -0.43 ± 0.91 | 0.58 ± 0.04 |
| Random | 150 | Cation Exchange Capacity | 0.48 ± 0.10 | 8.06 ± 2.81 | -0.05 ± 0.60 | 0.61 ± 0.11 | 0.41 ± 0.09 | 8.02 ± 0.93 | -0.17 ± 0.78 | 0.59 ± 0.06 |
| KS | 150 | Cation Exchange Capacity | 0.57* | 6.86* | 1.99* | 0.68* | 0.41* | 7.72* | 1.12* | 0.60* |
| cLHS | 150 | Cation Exchange Capacity | 0.50 ± 0.06 | 7.26 ± 0.52 | -0.15 ± 0.49 | 0.64 ± 0.04 | 0.43 ± 0.10 | 7.80 ± 0.98 | -0.49 ± 0.73 | 0.60 ± 0.07 |
| KM | 150 | Cation Exchange Capacity | 0.52 ± 0.07 | 6.95 ± 0.46 | 0.05 ± 0.51 | 0.67 ± 0.04 | 0.44 ± 0.07 | 7.59 ± 0.58 | -0.47 ± 0.71 | 0.62 ± 0.04 |
| Random | 200 | Cation Exchange Capacity | 0.51 ± 0.08 | 7.34 ± 1.21 | -0.11 ± 0.55 | 0.65 ± 0.08 | 0.43 ± 0.09 | 7.79 ± 0.88 | -0.38 ± 0.75 | 0.60 ± 0.06 |
| KS | 200 | Cation Exchange Capacity | 0.58* | 6.80* | 1.72* | 0.68* | 0.40* | 7.83* | 0.71* | 0.59* |
| cLHS | 200 | Cation Exchange Capacity | 0.51 ± 0.10 | 7.53 ± 2.22 | -0.15 ± 0.45 | 0.64 ± 0.09 | 0.45 ± 0.09 | 7.59 ± 0.77 | -0.32 ± 0.55 | 0.62 ± 0.06 |
| KM | 200 | Cation Exchange Capacity | 0.54 ± 0.06 | 6.83 ± 0.56 | 0.01 ± 0.37 | 0.68 ± 0.05 | 0.47 ± 0.09 | 7.40 ± 0.68 | -0.46 ± 0.48 | 0.63 ± 0.06 |
| Random | 250 | Cation Exchange Capacity | 0.54 ± 0.08 | 7.02 ± 1.15 | -0.14 ± 0.45 | 0.67 ± 0.08 | 0.45 ± 0.10 | 7.58 ± 0.94 | -0.35 ± 0.60 | 0.62 ± 0.07 |
| KS | 250 | Cation Exchange Capacity | 0.59* | 6.64* | 1.07* | 0.70* | 0.52* | 7.05* | -0.23* | 0.66* |
| cLHS | 250 | Cation Exchange Capacity | 0.55 ± 0.07 | 6.85 ± 0.86 | -0.16 ± 0.50 | 0.69 ± 0.07 | 0.46 ± 0.11 | 7.48 ± 0.83 | -0.44 ± 0.58 | 0.63 ± 0.07 |
| KM | 250 | Cation Exchange Capacity | 0.57 ± 0.05 | 6.62 ± 0.47 | -0.01 ± 0.30 | 0.71 ± 0.04 | 0.47 ± 0.10 | 7.39 ± 0.79 | -0.41 ± 0.68 | 0.64 ± 0.07 |
| Random | 300 | Cation Exchange Capacity | 0.54 ± 0.08 | 7.02 ± 1.20 | -0.12 ± 0.37 | 0.68 ± 0.09 | 0.49 ± 0.09 | 7.29 ± 0.72 | -0.33 ± 0.49 | 0.64 ± 0.06 |
| KS | 300 | Cation Exchange Capacity | 0.58* | 6.67* | 1.16* | 0.70* | 0.47* | 7.51* | -1.33* | 0.62* |
| cLHS | 300 | Cation Exchange Capacity | 0.55 ± 0.08 | 6.95 ± 1.71 | -0.13 ± 0.36 | 0.69 ± 0.08 | 0.46 ± 0.10 | 7.58 ± 0.99 | -0.36 ± 0.51 | 0.62 ± 0.07 |
| KM | 300 | Cation Exchange Capacity | 0.59 ± 0.03 | 6.43 ± 0.26 | 0.00 ± 0.36 | 0.72 ± 0.03 | 0.44 ± 0.09 | 7.53 ± 0.72 | -0.49 ± 0.46 | 0.62 ± 0.06 |
| Random | 400 | Cation Exchange Capacity | 0.58 ± 0.06 | 6.60 ± 0.77 | -0.16 ± 0.33 | 0.71 ± 0.06 | 0.49 ± 0.08 | 7.25 ± 0.74 | -0.34 ± 0.39 | 0.65 ± 0.06 |
| KS | 400 | Cation Exchange Capacity | 0.60* | 6.55* | 1.11* | 0.71* | 0.39* | 7.77* | 0.06* | 0.60* |
| cLHS | 400 | Cation Exchange Capacity | 0.57 ± 0.09 | 6.73 ± 1.35 | -0.23 ± 0.27 | 0.71 ± 0.08 | 0.50 ± 0.07 | 7.14 ± 0.58 | -0.51 ± 0.36 | 0.66 ± 0.05 |
| KM | 400 | Cation Exchange Capacity | 0.60 ± 0.04 | 6.32 ± 0.30 | 0.05 ± 0.26 | 0.74 ± 0.03 | 0.49 ± 0.06 | 7.20 ± 0.47 | -0.36 ± 0.35 | 0.65 ± 0.04 |
| Random | 500 | Cation Exchange Capacity | 0.59 ± 0.05 | 6.43 ± 0.55 | -0.21 ± 0.29 | 0.73 ± 0.05 | 0.50 ± 0.07 | 7.17 ± 0.64 | -0.39 ± 0.38 | 0.65 ± 0.05 |
| KS | 500 | Cation Exchange Capacity | 0.60* | 6.56* | 1.37* | 0.71* | 0.46* | 7.42* | 0.96* | 0.63* |
| cLHS | 500 | Cation Exchange Capacity | 0.60 ± 0.05 | 6.35 ± 0.51 | -0.17 ± 0.26 | 0.74 ± 0.05 | 0.51 ± 0.08 | 7.07 ± 0.63 | -0.41 ± 0.37 | 0.66 ± 0.06 |
| KM | 500 | Cation Exchange Capacity | 0.62 ± 0.03 | 6.17 ± 0.30 | -0.02 ± 0.22 | 0.75 ± 0.03 | 0.48 ± 0.08 | 7.30 ± 0.71 | -0.47 ± 0.34 | 0.64 ± 0.06 |
| Random | 1000 | Cation Exchange Capacity | 0.64 ± 0.02 | 5.95 ± 0.20 | -0.20 ± 0.18 | 0.78 ± 0.03 | 0.54 ± 0.06 | 6.79 ± 0.49 | -0.44 ± 0.27 | 0.69 ± 0.05 |
| KS | 1000 | Cation Exchange Capacity | 0.64* | 6.07* | 0.60* | 0.77* | 0.43* | 7.58* | -0.96* | 0.61* |
| cLHS | 1000 | Cation Exchange Capacity | 0.65 ± 0.02 | 5.88 ± 0.19 | -0.20 ± 0.14 | 0.79 ± 0.02 | 0.55 ± 0.06 | 6.73 ± 0.46 | -0.48 ± 0.26 | 0.69 ± 0.05 |
| KM | 1000 | Cation Exchange Capacity | 0.65 ± 0.03 | 5.93 ± 0.27 | -0.12 ± 0.15 | 0.79 ± 0.03 | 0.54 ± 0.05 | 6.83 ± 0.37 | -0.44 ± 0.26 | 0.68 ± 0.04 |
| Random | 1500 | Cation Exchange Capacity | 0.67 ± 0.01 | 5.75 ± 0.10 | -0.20 ± 0.12 | 0.81 ± 0.01 | 0.57 ± 0.04 | 6.59 ± 0.33 | -0.48 ± 0.23 | 0.71 ± 0.03 |
| KS | 1500 | Cation Exchange Capacity | 0.66* | 5.92* | 0.30* | 0.79* | 0.56* | 6.65* | -0.52* | 0.70* |
| cLHS | 1500 | Cation Exchange Capacity | 0.67 ± 0.01 | 5.73 ± 0.08 | -0.19 ± 0.12 | 0.81 ± 0.01 | 0.58 ± 0.05 | 6.53 ± 0.38 | -0.51 ± 0.22 | 0.71 ± 0.04 |
| KM | 1500 | Cation Exchange Capacity | 0.66 ± 0.02 | 5.77 ± 0.14 | -0.17 ± 0.16 | 0.81 ± 0.02 | 0.56 ± 0.05 | 6.66 ± 0.40 | -0.48 ± 0.24 | 0.70 ± 0.04 |
| Random | 2000 | Cation Exchange Capacity | 0.67 ± 0.01 | 5.69 ± 0.06 | -0.19 ± 0.10 | 0.82 ± 0.01 | 0.58 ± 0.04 | 6.47 ± 0.34 | -0.52 ± 0.25 | 0.72 ± 0.04 |
| KS | 2000 | Cation Exchange Capacity | 0.67* | 5.79* | 0.31* | 0.80* | 0.58* | 6.45* | 0.13* | 0.72* |
| cLHS | 2000 | Cation Exchange Capacity | 0.67 ± 0.01 | 5.69 ± 0.06 | -0.24 ± 0.10 | 0.82 ± 0.01 | 0.59 ± 0.04 | 6.39 ± 0.31 | -0.56 ± 0.18 | 0.73 ± 0.04 |
| KM | 2000 | Cation Exchange Capacity | 0.67 ± 0.02 | 5.72 ± 0.15 | -0.25 ± 0.16 | 0.81 ± 0.02 | 0.56 ± 0.05 | 6.69 ± 0.44 | -0.50 ± 0.25 | 0.70 ± 0.05 |
| Random | 3000 | Cation Exchange Capacity | 0.68 ± 0.00 | 5.63 ± 0.03 | -0.20 ± 0.06 | 0.83 ± 0.00 | 0.61 ± 0.03 | 6.30 ± 0.27 | -0.50 ± 0.17 | 0.74 ± 0.03 |
| KS | 3000 | Cation Exchange Capacity | 0.68* | 5.66* | 0.11* | 0.82* | 0.62* | 6.16* | -0.51* | 0.76* |
| cLHS | 3000 | Cation Exchange Capacity | 0.68 ± 0.00 | 5.64 ± 0.03 | -0.22 ± 0.06 | 0.82 ± 0.01 | 0.61 ± 0.03 | 6.25 ± 0.30 | -0.50 ± 0.19 | 0.75 ± 0.03 |
| KM | 3000 | Cation Exchange Capacity | 0.66 ± 0.04 | 5.84 ± 0.37 | -0.27 ± 0.17 | 0.80 ± 0.04 | 0.48 ± 0.12 | 7.65 ± 1.30 | -0.38 ± 0.28 | 0.62 ± 0.10 |

*Only represents one repetition. RPIQ: Ratio of Performance to Interquartile distance, which can be calculated as RPIQ=IQ/RMSE where IQ = Q3-Q1; IQ being the interquartile distance of the validation set, Q1 the median of the first half of the validation set and Q3 the median for the second half of the validation set.

# Appendix B. Summary of model performance to predict various soil properties (clay content, sand content, total carbon, pH and cation exchange capacity) using two different regression models (Partial Least Square Regression (PLSR) and Cubist) with various sampling algorithms (Random, Kennard-Stone (KS), conditioned Latin Hypercube sampling (cLHS), k-Means(KM)) and calibration sample sizes (50-200) in the regional dataset. The results reported are averages and standard deviations from 50 repetitions.

| Sampling Algorithm | Calibration sample size | Soil Property | PLSR | | | | Cubist | | | |
| --- | --- | --- | --- | --- | --- | --- | --- | --- | --- | --- |
|  |  |  | R^2^ | RMSE | bias | RPIQ | R^2^ | RMSE | bias | RPIQ |
| Random | 50 | clay | 0.74 ± 0.08 | 9.04 ± 1.53 | -0.03 ± 2.20 | 0.45 ± 0.07 | 0.70 ± 0.08 | 9.82 ± 1.59 | -0.35 ± 2.47 | 0.42 ± 0.06 |
| cLHS | 50 | clay | 0.73 ± 0.09 | 9.21 ± 1.54 | -0.59 ± 1.88 | 0.44 ± 0.07 | 0.69 ± 0.09 | 9.88 ± 1.63 | -0.86 ± 2.12 | 0.42 ± 0.07 |
| KM | 50 | clay | 0.71 ± 0.14 | 9.45 ± 2.27 | -1.15 ± 1.70 | 0.44 ± 0.07 | 0.67 ± 0.11 | 10.13 ± 1.80 | -1.81 ± 1.62 | 0.41 ± 0.07 |
| KS | 50 | clay | 0.78* | 8.59* | -1.33* | 0.47* | 0.71* | 9.83* | -0.48* | 0.41* |
| Random | 100 | clay | 0.73 ± 0.11 | 9.14 ± 1.94 | -0.24 ± 1.52 | 0.45 ± 0.07 | 0.72 ± 0.08 | 9.30 ± 1.41 | -0.71 ± 1.57 | 0.44 ± 0.06 |
| cLHS | 100 | clay | 0.73 ± 0.09 | 9.08 ± 1.48 | -0.41 ± 1.64 | 0.45 ± 0.06 | 0.66 ± 0.14 | 10.12 ± 2.22 | -0.80 ± 1.74 | 0.41 ± 0.07 |
| KM | 100 | clay | 0.75 ± 0.06 | 8.75 ± 1.13 | -0.05 ± 0.99 | 0.46 ± 0.05 | 0.67 ± 0.10 | 10.02 ± 1.69 | -0.41 ± 1.61 | 0.41 ± 0.06 |
| KS | 100 | clay | 0.68* | 9.94* | -2.16* | 0.40* | 0.68* | 10.32* | -3.47* | 0.39* |
| Random | 150 | clay | 0.74 ± 0.06 | 8.83 ± 1.08 | -0.31 ± 1.07 | 0.46 ± 0.05 | 0.71 ± 0.08 | 9.35 ± 1.30 | -0.15 ± 1.33 | 0.44 ± 0.05 |
| cLHS | 150 | clay | 0.75 ± 0.04 | 8.60 ± 0.71 | -0.48 ± 0.81 | 0.47 ± 0.04 | 0.73 ± 0.07 | 9.00 ± 1.09 | -0.44 ± 0.98 | 0.45 ± 0.05 |
| KM | 150 | clay | 0.74 ± 0.06 | 8.85 ± 1.00 | -0.18 ± 1.20 | 0.46 ± 0.05 | 0.65 ± 0.11 | 10.47 ± 1.78 | 0.06 ± 1.59 | 0.39 ± 0.06 |
| KS | 150 | clay | 0.75* | 8.67* | -0.75* | 0.46* | 0.70* | 9.65* | -2.09* | 0.41* |
| Random | 200 | clay | 0.75 ± 0.03 | 8.62 ± 0.51 | -0.10 ± 0.81 | 0.47 ± 0.03 | 0.72 ± 0.08 | 9.15 ± 1.19 | -0.13 ± 1.00 | 0.44 ± 0.05 |
| cLHS | 200 | clay | 0.74 ± 0.03 | 8.72 ± 0.53 | -0.47 ± 0.72 | 0.46 ± 0.03 | 0.72 ± 0.08 | 9.20 ± 1.32 | 0.00 ± 0.97 | 0.44 ± 0.06 |
| KM | 200 | clay | 0.68 ± 0.08 | 10.12 ± 1.63 | -0.02 ± 1.64 | 0.41 ± 0.07 | 0.60 ± 0.14 | 11.32 ± 2.50 | -0.02 ± 1.69 | 0.37 ± 0.07 |
| KS | 200 | clay | 0.75* | 8.63* | -0.39* | 0.46* | 0.78* | 8.10* | 0.40* | 0.49* |
| Random | 50 | sand | 0.69 ± 0.11 | 9.95 ± 2.23 | -1.45 ± 2.43 | 1.46 ± 0.24 | 0.69 ± 0.06 | 9.54 ± 1.10 | -0.72 ± 2.01 | 1.49 ± 0.16 |
| cLHS | 50 | sand | 0.70 ± 0.09 | 9.43 ± 1.53 | -1.37 ± 1.82 | 1.52 ± 0.20 | 0.68 ± 0.10 | 9.47 ± 1.40 | -0.65 ± 1.59 | 1.51 ± 0.20 |
| KM | 50 | sand | 0.65 ± 0.11 | 9.98 ± 1.94 | -0.61 ± 2.23 | 1.45 ± 0.23 | 0.66 ± 0.10 | 9.68 ± 1.53 | -0.16 ± 2.00 | 1.48 ± 0.20 |
| KS | 50 | sand | 0.74* | 8.35* | 0.58* | 1.68* | 0.74* | 8.40* | -0.63* | 1.67* |
| Random | 100 | sand | 0.71 ± 0.05 | 9.14 ± 1.11 | -1.70 ± 1.26 | 1.55 ± 0.16 | 0.69 ± 0.08 | 9.39 ± 1.18 | -1.20 ± 1.67 | 1.51 ± 0.17 |
| cLHS | 100 | sand | 0.72 ± 0.05 | 9.00 ± 0.83 | -1.51 ± 1.21 | 1.57 ± 0.12 | 0.69 ± 0.08 | 9.42 ± 1.22 | -1.00 ± 1.47 | 1.51 ± 0.17 |
| KM | 100 | sand | 0.70 ± 0.07 | 9.26 ± 1.15 | -1.70 ± 1.30 | 1.53 ± 0.17 | 0.64 ± 0.11 | 10.06 ± 1.62 | -1.14 ± 1.52 | 1.43 ± 0.22 |
| KS | 100 | sand | 0.74* | 8.42* | -0.83* | 1.66* | 0.72* | 8.75* | 1.21* | 1.60* |
| Random | 150 | sand | 0.72 ± 0.05 | 8.87 ± 0.79 | -1.84 ± 0.88 | 1.59 ± 0.13 | 0.68 ± 0.09 | 9.65 ± 1.42 | -1.71 ± 1.59 | 1.48 ± 0.19 |
| cLHS | 150 | sand | 0.74 ± 0.03 | 8.62 ± 0.52 | -1.87 ± 0.78 | 1.63 ± 0.09 | 0.68 ± 0.07 | 9.47 ± 1.21 | -1.55 ± 1.06 | 1.50 ± 0.17 |
| KM | 150 | sand | 0.70 ± 0.08 | 9.45 ± 1.28 | -2.38 ± 1.22 | 1.51 ± 0.19 | 0.63 ± 0.12 | 10.85 ± 2.37 | -2.30 ± 1.81 | 1.33 ± 0.22 |
| KS | 150 | sand | 0.75* | 8.23* | -0.84* | 1.70* | 0.75* | 8.32* | -0.06* | 1.68* |
| Random | 200 | sand | 0.74 ± 0.02 | 8.58 ± 0.33 | -1.98 ± 0.49 | 1.63 ± 0.06 | 0.71 ± 0.05 | 9.11 ± 0.86 | -2.00 ± 1.02 | 1.55 ± 0.14 |
| cLHS | 200 | sand | 0.74 ± 0.03 | 8.49 ± 0.37 | -1.71 ± 0.62 | 1.65 ± 0.07 | 0.71 ± 0.05 | 9.14 ± 0.84 | -1.96 ± 1.04 | 1.54 ± 0.13 |
| KM | 200 | sand | 0.62 ± 0.14 | 11.12 ± 2.34 | -1.68 ± 2.00 | 1.31 ± 0.27 | 0.53 ± 0.14 | 12.80 ± 2.91 | -2.07 ± 2.54 | 1.14 ± 0.24 |
| KS | 200 | sand | 0.77* | 7.98* | -1.52* | 1.75* | 0.76* | 8.04* | -1.12* | 1.74* |
| Random | 50 | Total Carbon | 0.63 ± 0.10 | 0.62 ± 0.17 | -0.08 ± 0.13 | 1.28 ± 0.24 | 0.62 ± 0.09 | 0.61 ± 0.12 | -0.06 ± 0.11 | 1.28 ± 0.23 |
| cLHS | 50 | Total Carbon | 0.66 ± 0.07 | 0.57 ± 0.09 | -0.07 ± 0.08 | 1.36 ± 0.19 | 0.65 ± 0.06 | 0.57 ± 0.08 | -0.05 ± 0.10 | 1.35 ± 0.18 |
| KM | 50 | Total Carbon | 0.65 ± 0.08 | 0.60 ± 0.13 | -0.10 ± 0.10 | 1.31 ± 0.23 | 0.61 ± 0.08 | 0.63 ± 0.12 | -0.07 ± 0.12 | 1.25 ± 0.20 |
| KS | 50 | Total Carbon | 0.70* | 0.50* | -0.12* | 1.51* | 0.60* | 0.61* | -0.03* | 1.25* |
| Random | 100 | Total Carbon | 0.69 ± 0.04 | 0.54 ± 0.06 | -0.04 ± 0.07 | 1.44 ± 0.16 | 0.66 ± 0.06 | 0.56 ± 0.08 | -0.05 ± 0.09 | 1.37 ± 0.18 |
| cLHS | 100 | Total Carbon | 0.69 ± 0.04 | 0.53 ± 0.06 | -0.03 ± 0.08 | 1.45 ± 0.15 | 0.68 ± 0.06 | 0.54 ± 0.06 | -0.05 ± 0.07 | 1.42 ± 0.17 |
| KM | 100 | Total Carbon | 0.69 ± 0.05 | 0.53 ± 0.06 | -0.07 ± 0.05 | 1.44 ± 0.15 | 0.66 ± 0.05 | 0.57 ± 0.06 | -0.06 ± 0.07 | 1.35 ± 0.13 |
| KS | 100 | Total Carbon | 0.73* | 0.48* | -0.12* | 1.59* | 0.67* | 0.53* | -0.05* | 1.43* |
| Random | 150 | Total Carbon | 0.71 ± 0.04 | 0.51 ± 0.04 | -0.03 ± 0.06 | 1.51 ± 0.13 | 0.69 ± 0.04 | 0.53 ± 0.05 | -0.05 ± 0.06 | 1.44 ± 0.14 |
| cLHS | 150 | Total Carbon | 0.71 ± 0.03 | 0.49 ± 0.04 | -0.01 ± 0.04 | 1.54 ± 0.11 | 0.70 ± 0.05 | 0.52 ± 0.05 | -0.03 ± 0.05 | 1.49 ± 0.15 |
| KM | 150 | Total Carbon | 0.71 ± 0.05 | 0.54 ± 0.07 | -0.03 ± 0.08 | 1.44 ± 0.17 | 0.64 ± 0.08 | 0.59 ± 0.10 | -0.05 ± 0.07 | 1.32 ± 0.21 |
| KS | 150 | Total Carbon | 0.76* | 0.45* | -0.06* | 1.69* | 0.75* | 0.47* | -0.06* | 1.61* |
| Random | 200 | Total Carbon | 0.72 ± 0.02 | 0.49 ± 0.03 | -0.01 ± 0.04 | 1.57 ± 0.09 | 0.70 ± 0.04 | 0.51 ± 0.04 | -0.03 ± 0.05 | 1.49 ± 0.13 |
| cLHS | 200 | Total Carbon | 0.73 ± 0.03 | 0.49 ± 0.03 | -0.02 ± 0.05 | 1.57 ± 0.10 | 0.70 ± 0.04 | 0.52 ± 0.04 | -0.05 ± 0.04 | 1.47 ± 0.11 |
| KM | 200 | Total Carbon | 0.72 ± 0.04 | 0.55 ± 0.07 | 0.01 ± 0.10 | 1.40 ± 0.17 | 0.59 ± 0.13 | 0.67 ± 0.19 | -0.05 ± 0.10 | 1.20 ± 0.28 |
| KS | 200 | Total Carbon | 0.74* | 0.47* | -0.05* | 1.62* | 0.72* | 0.50* | -0.04* | 1.53* |
| Random | 50 | pH.in.CaCl2 | 0.62 ± 0.08 | 0.76 ± 0.09 | -0.01 ± 0.15 | 0.93 ± 0.11 | 0.59 ± 0.09 | 0.78 ± 0.09 | -0.04 ± 0.13 | 0.90 ± 0.10 |
| cLHS | 50 | pH.in.CaCl2 | 0.65 ± 0.07 | 0.76 ± 0.11 | 0.04 ± 0.16 | 0.93 ± 0.12 | 0.59 ± 0.10 | 0.78 ± 0.09 | -0.04 ± 0.14 | 0.90 ± 0.10 |
| KM | 50 | pH.in.CaCl2 | 0.63 ± 0.10 | 0.79 ± 0.13 | 0.13 ± 0.14 | 0.91 ± 0.13 | 0.57 ± 0.10 | 0.80 ± 0.11 | 0.02 ± 0.15 | 0.88 ± 0.11 |
| KS | 50 | pH.in.CaCl2 | 0.66* | 0.70* | 0.08* | 0.99* | 0.72* | 0.62* | -0.01* | 1.13* |
| Random | 100 | pH.in.CaCl2 | 0.71 ± 0.05 | 0.65 ± 0.07 | 0.04 ± 0.12 | 1.08 ± 0.11 | 0.66 ± 0.05 | 0.69 ± 0.06 | 0.02 ± 0.09 | 1.01 ± 0.08 |
| cLHS | 100 | pH.in.CaCl2 | 0.70 ± 0.05 | 0.66 ± 0.06 | 0.05 ± 0.10 | 1.05 ± 0.09 | 0.62 ± 0.11 | 0.74 ± 0.12 | 0.01 ± 0.10 | 0.96 ± 0.12 |
| KM | 100 | pH.in.CaCl2 | 0.69 ± 0.08 | 0.69 ± 0.09 | 0.10 ± 0.10 | 1.02 ± 0.12 | 0.64 ± 0.11 | 0.73 ± 0.12 | 0.08 ± 0.11 | 0.97 ± 0.12 |
| KS | 100 | pH.in.CaCl2 | 0.71* | 0.64* | 0.13* | 1.09* | 0.61* | 0.74* | -0.07* | 0.95* |
| Random | 150 | pH.in.CaCl2 | 0.76 ± 0.04 | 0.59 ± 0.06 | 0.05 ± 0.08 | 1.18 ± 0.11 | 0.70 ± 0.07 | 0.65 ± 0.08 | 0.04 ± 0.06 | 1.08 ± 0.12 |
| cLHS | 150 | pH.in.CaCl2 | 0.75 ± 0.05 | 0.60 ± 0.07 | 0.06 ± 0.08 | 1.17 ± 0.13 | 0.69 ± 0.07 | 0.65 ± 0.08 | 0.04 ± 0.08 | 1.08 ± 0.12 |
| KM | 150 | pH.in.CaCl2 | 0.71 ± 0.06 | 0.68 ± 0.09 | 0.09 ± 0.13 | 1.05 ± 0.14 | 0.63 ± 0.12 | 0.75 ± 0.14 | 0.08 ± 0.13 | 0.95 ± 0.16 |
| KS | 150 | pH.in.CaCl2 | 0.71* | 0.64* | 0.14* | 1.08* | 0.74* | 0.60* | 0.07* | 1.16* |
| Random | 200 | pH.in.CaCl2 | 0.79 ± 0.02 | 0.55 ± 0.04 | 0.04 ± 0.06 | 1.26 ± 0.08 | 0.73 ± 0.06 | 0.62 ± 0.08 | 0.04 ± 0.08 | 1.14 ± 0.13 |
| cLHS | 200 | pH.in.CaCl2 | 0.77 ± 0.03 | 0.57 ± 0.04 | 0.05 ± 0.06 | 1.22 ± 0.08 | 0.73 ± 0.04 | 0.62 ± 0.05 | 0.00 ± 0.07 | 1.13 ± 0.09 |
| KM | 200 | pH.in.CaCl2 | 0.68 ± 0.10 | 0.71 ± 0.15 | 0.11 ± 0.13 | 1.02 ± 0.19 | 0.52 ± 0.20 | 0.86 ± 0.23 | 0.07 ± 0.13 | 0.86 ± 0.22 |
| KS | 200 | pH.in.CaCl2 | 0.79* | 0.54* | 0.04* | 1.28* | 0.70* | 0.65* | 0.07* | 1.07* |
| Random | 50 | Cation Exchange Capacity | 0.77 ± 0.03 | 3.52 ± 0.25 | 0.46 ± 0.51 | 0.97 ± 0.07 | 0.74 ± 0.05 | 3.84 ± 0.43 | 0.32 ± 0.85 | 0.90 ± 0.09 |
| cLHS | 50 | Cation Exchange Capacity | 0.78 ± 0.02 | 3.57 ± 0.33 | 0.70 ± 0.52 | 0.96 ± 0.08 | 0.74 ± 0.08 | 3.83 ± 0.54 | 0.44 ± 0.68 | 0.90 ± 0.11 |
| KM | 50 | Cation Exchange Capacity | 0.78 ± 0.03 | 3.57 ± 0.36 | 0.53 ± 0.61 | 0.96 ± 0.09 | 0.73 ± 0.06 | 3.87 ± 0.48 | 0.22 ± 0.76 | 0.89 ± 0.10 |
| KS | 50 | Cation Exchange Capacity | 0.76* | 4.51* | 2.31* | 0.75* | 0.75* | 3.97* | 1.31* | 0.86* |
| Random | 100 | Cation Exchange Capacity | 0.80 ± 0.02 | 3.32 ± 0.18 | 0.63 ± 0.36 | 1.03 ± 0.06 | 0.78 ± 0.02 | 3.49 ± 0.27 | 0.60 ± 0.46 | 0.98 ± 0.07 |
| cLHS | 100 | Cation Exchange Capacity | 0.80 ± 0.02 | 3.31 ± 0.14 | 0.56 ± 0.32 | 1.03 ± 0.05 | 0.77 ± 0.04 | 3.53 ± 0.26 | 0.42 ± 0.47 | 0.97 ± 0.07 |
| KM | 100 | Cation Exchange Capacity | 0.80 ± 0.02 | 3.29 ± 0.22 | 0.61 ± 0.32 | 1.04 ± 0.07 | 0.76 ± 0.06 | 3.59 ± 0.44 | 0.59 ± 0.47 | 0.96 ± 0.10 |
| KS | 100 | Cation Exchange Capacity | 0.83* | 3.10* | 0.84* | 1.10* | 0.80* | 3.48* | 1.34* | 0.98* |
| Random | 150 | Cation Exchange Capacity | 0.81 ± 0.01 | 3.21 ± 0.13 | 0.64 ± 0.22 | 1.06 ± 0.04 | 0.79 ± 0.02 | 3.41 ± 0.21 | 0.64 ± 0.32 | 1.00 ± 0.06 |
| cLHS | 150 | Cation Exchange Capacity | 0.81 ± 0.01 | 3.21 ± 0.12 | 0.58 ± 0.21 | 1.06 ± 0.04 | 0.79 ± 0.04 | 3.40 ± 0.29 | 0.55 ± 0.38 | 1.01 ± 0.07 |
| KM | 150 | Cation Exchange Capacity | 0.80 ± 0.03 | 3.33 ± 0.26 | 0.47 ± 0.38 | 1.03 ± 0.08 | 0.75 ± 0.07 | 3.68 ± 0.54 | 0.57 ± 0.44 | 0.94 ± 0.12 |
| KS | 150 | Cation Exchange Capacity | 0.82* | 3.16* | 0.73* | 1.08* | 0.77* | 3.51* | 0.71* | 0.97* |
| Random | 200 | Cation Exchange Capacity | 0.82 ± 0.01 | 3.15 ± 0.12 | 0.61 ± 0.21 | 1.08 ± 0.04 | 0.80 ± 0.01 | 3.33 ± 0.13 | 0.62 ± 0.28 | 1.02 ± 0.04 |
| cLHS | 200 | Cation Exchange Capacity | 0.82 ± 0.01 | 3.13 ± 0.09 | 0.51 ± 0.16 | 1.09 ± 0.03 | 0.80 ± 0.02 | 3.28 ± 0.15 | 0.51 ± 0.28 | 1.04 ± 0.05 |
| KM | 200 | Cation Exchange Capacity | 0.78 ± 0.04 | 3.44 ± 0.31 | 0.38 ± 0.47 | 0.99 ± 0.09 | 0.68 ± 0.12 | 4.22 ± 0.94 | 0.54 ± 0.44 | 0.84 ± 0.16 |
| KS | 200 | Cation Exchange Capacity | 0.82* | 3.14* | 0.67* | 1.08* | 0.82* | 3.06* | 0.31* | 1.11* |

*Only represents one repetition. RPIQ: Ratio of Performance to Interquartile distance, which can be calculated as RPIQ=IQ/RMSE where IQ = Q3-Q1; IQ being the interquartile distance of the validation set, Q1 the median of the first half of the validation set and Q3 the median for the second half of the validation set.

# Appendix C. Summary of model performance to predict various soil properties (clay content, sand content, pH and cation exchange capacity) using two different regression models (Partial Least Square Regression (PLSR) and Cubist) with various sampling algorithms (Random, Kennard-Stone (KS), conditioned Latin Hypercube sampling (cLHS), k-Means(KM)) and calibration sample sizes (50-200) in the local dataset. The results reported are averages and standard deviations from 50 repetitions.

| Sampling Algorithm | Calibration sample size | Soil Property | PLSR | | | |  | Cubist | | |
| --- | --- | --- | --- | --- | --- | --- | --- | --- | --- | --- |
|  |  |  | R^2^ | RMSE | bias | RPIQ | R^2^ | RMSE | bias | RPIQ |
| Random | 50 | clay | 0.67 ± 0.07 | 8.04 ± 0.85 | 1.96 ± 1.30 | 1.02 ± 0.11 | 0.66 ± 0.07 | 8.44 ± 0.96 | 3.07 ± 1.29 | 0.97 ± 0.11 |
| KS | 50 | clay | 0.62* | 8.41* | 2.32* | 0.97* | 0.72* | 6.93* | 1.06* | 1.17* |
| CLHS | 50 | clay | 0.68 ± 0.07 | 8.02 ± 0.93 | 2.52 ± 0.94 | 1.03 ± 0.12 | 0.66 ± 0.07 | 8.45 ± 0.80 | 3.36 ± 1.00 | 0.97 ± 0.09 |
| KM | 50 | clay | 0.67 ± 0.07 | 8.02 ± 0.73 | 2.23 ± 1.17 | 1.02 ± 0.09 | 0.65 ± 0.10 | 8.58 ± 1.40 | 3.17 ± 1.19 | 0.97 ± 0.13 |
| Random | 100 | clay | 0.74 ± 0.04 | 6.99 ± 0.47 | 1.90 ± 0.59 | 1.17 ± 0.08 | 0.70 ± 0.07 | 7.75 ± 0.84 | 2.85 ± 0.78 | 1.06 ± 0.11 |
| KS | 100 | clay | 0.72* | 7.39* | 2.49* | 1.10* | 0.72* | 7.35* | 2.65* | 1.10* |
| CLHS | 100 | clay | 0.73 ± 0.05 | 7.09 ± 0.62 | 2.11 ± 0.66 | 1.16 ± 0.10 | 0.70 ± 0.07 | 7.75 ± 0.89 | 3.02 ± 0.65 | 1.06 ± 0.12 |
| KM | 100 | clay | 0.73 ± 0.04 | 7.23 ± 0.50 | 2.17 ± 0.74 | 1.13 ± 0.08 | 0.67 ± 0.08 | 8.23 ± 1.04 | 3.13 ± 0.95 | 1.00 ± 0.13 |
| Random | 150 | clay | 0.76 ± 0.02 | 6.66 ± 0.35 | 1.89 ± 0.44 | 1.22 ± 0.07 | 0.71 ± 0.08 | 7.56 ± 0.92 | 2.77 ± 0.49 | 1.09 ± 0.11 |
| KS | 150 | clay | 0.76* | 6.76* | 2.19* | 1.20* | 0.75* | 7.15* | 2.91* | 1.14* |
| CLHS | 150 | clay | 0.75 ± 0.03 | 6.82 ± 0.44 | 1.99 ± 0.31 | 1.20 ± 0.08 | 0.71 ± 0.09 | 7.63 ± 1.35 | 2.84 ± 0.62 | 1.09 ± 0.12 |
| KM | 150 | clay | 0.75 ± 0.04 | 6.90 ± 0.59 | 2.04 ± 0.75 | 1.19 ± 0.10 | 0.68 ± 0.09 | 8.03 ± 1.21 | 2.83 ± 0.93 | 1.03 ± 0.15 |
| Random | 200 | clay | 0.77 ± 0.03 | 6.53 ± 0.38 | 1.88 ± 0.31 | 1.25 ± 0.07 | 0.73 ± 0.05 | 7.34 ± 0.64 | 2.77 ± 0.45 | 1.12 ± 0.09 |
| KS | 200 | clay | 0.79* | 6.20* | 1.49* | 1.31* | 0.70* | 7.77* | 2.75* | 1.05* |
| CLHS | 200 | clay | 0.77 ± 0.02 | 6.60 ± 0.30 | 2.00 ± 0.30 | 1.23 ± 0.06 | 0.74 ± 0.05 | 7.18 ± 0.60 | 2.63 ± 0.41 | 1.14 ± 0.09 |
| KM | 200 | clay | 0.73 ± 0.06 | 7.07 ± 0.79 | 2.04 ± 0.75 | 1.16 ± 0.12 | 0.63 ± 0.15 | 8.54 ± 1.87 | 3.03 ± 1.04 | 0.99 ± 0.19 |
| Random | 50 | sand | 0.61 ± 0.10 | 9.31 ± 1.24 | -2.08 ± 1.60 | 0.56 ± 0.07 | 0.62 ± 0.09 | 9.38 ± 1.29 | -3.30 ± 1.46 | 0.55 ± 0.07 |
| KS | 50 | sand | 0.54* | 9.55* | -2.36* | 0.54* | 0.64* | 8.40* | -2.43* | 0.60* |
| CLHS | 50 | sand | 0.61 ± 0.10 | 9.29 ± 1.41 | -2.44 ± 1.36 | 0.56 ± 0.07 | 0.63 ± 0.11 | 9.22 ± 1.33 | -3.18 ± 1.45 | 0.56 ± 0.08 |
| KM | 50 | sand | 0.59 ± 0.13 | 9.48 ± 1.77 | -2.42 ± 1.81 | 0.55 ± 0.08 | 0.65 ± 0.08 | 9.05 ± 1.21 | -2.80 ± 1.71 | 0.57 ± 0.07 |
| Random | 100 | sand | 0.68 ± 0.06 | 8.04 ± 0.75 | -2.04 ± 0.89 | 0.64 ± 0.06 | 0.67 ± 0.07 | 8.28 ± 0.95 | -2.55 ± 0.95 | 0.62 ± 0.07 |
| KS | 100 | sand | 0.63* | 9.09* | -3.49* | 0.56* | 0.69* | 8.38* | -3.77* | 0.61* |
| CLHS | 100 | sand | 0.65 ± 0.07 | 8.45 ± 0.83 | -2.31 ± 0.90 | 0.61 ± 0.06 | 0.66 ± 0.09 | 8.42 ± 1.10 | -2.73 ± 0.85 | 0.61 ± 0.08 |
| KM | 100 | sand | 0.63 ± 0.07 | 8.71 ± 1.03 | -2.41 ± 1.46 | 0.59 ± 0.07 | 0.61 ± 0.11 | 9.10 ± 1.41 | -2.74 ± 1.24 | 0.57 ± 0.09 |
| Random | 150 | sand | 0.69 ± 0.04 | 7.85 ± 0.51 | -1.99 ± 0.70 | 0.65 ± 0.04 | 0.68 ± 0.08 | 8.21 ± 1.16 | -2.56 ± 0.91 | 0.63 ± 0.08 |
| KS | 150 | sand | 0.66* | 8.52* | -3.27* | 0.60* | 0.77* | 7.03* | -2.83* | 0.72* |
| CLHS | 150 | sand | 0.68 ± 0.03 | 7.94 ± 0.43 | -2.00 ± 0.46 | 0.64 ± 0.04 | 0.68 ± 0.07 | 8.06 ± 0.93 | -2.37 ± 0.78 | 0.64 ± 0.08 |
| KM | 150 | sand | 0.64 ± 0.07 | 8.51 ± 1.00 | -2.26 ± 1.52 | 0.60 ± 0.07 | 0.60 ± 0.11 | 9.22 ± 1.57 | -2.82 ± 1.57 | 0.57 ± 0.10 |
| Random | 200 | sand | 0.70 ± 0.04 | 7.61 ± 0.50 | -1.85 ± 0.48 | 0.67 ± 0.04 | 0.68 ± 0.07 | 8.05 ± 0.89 | -2.45 ± 0.73 | 0.64 ± 0.07 |
| KS | 200 | sand | 0.66* | 8.26* | -2.66* | 0.62* | 0.67* | 8.25* | -2.93* | 0.62* |
| CLHS | 200 | sand | 0.70 ± 0.03 | 7.74 ± 0.40 | -2.06 ± 0.42 | 0.66 ± 0.03 | 0.69 ± 0.07 | 8.03 ± 0.99 | -2.51 ± 0.59 | 0.64 ± 0.07 |
| KM | 200 | sand | 0.62 ± 0.09 | 8.72 ± 1.20 | -2.42 ± 1.31 | 0.59 ± 0.08 | 0.52 ± 0.19 | 10.13 ± 2.63 | -3.13 ± 1.48 | 0.53 ± 0.13 |
| Random | 50 | pH | 0.57 ± 0.11 | 0.60 ± 0.10 | 0.04 ± 0.10 | 1.19 ± 0.17 | 0.45 ± 0.12 | 0.66 ± 0.09 | 0.00 ± 0.12 | 1.07 ± 0.14 |
| KS | 50 | pH | 0.58* | 0.56* | 0.00* | 1.25* | 0.51* | 0.60* | 0.02* | 1.16* |
| CLHS | 50 | pH | 0.58 ± 0.10 | 0.59 ± 0.09 | 0.01 ± 0.11 | 1.21 ± 0.18 | 0.46 ± 0.14 | 0.66 ± 0.10 | 0.02 ± 0.14 | 1.08 ± 0.18 |
| KM | 50 | pH | 0.60 ± 0.07 | 0.59 ± 0.09 | 0.00 ± 0.11 | 1.20 ± 0.18 | 0.48 ± 0.13 | 0.65 ± 0.14 | 0.00 ± 0.10 | 1.10 ± 0.18 |
| Random | 100 | pH | 0.65 ± 0.08 | 0.52 ± 0.07 | 0.01 ± 0.06 | 1.35 ± 0.17 | 0.63 ± 0.07 | 0.53 ± 0.05 | 0.04 ± 0.07 | 1.34 ± 0.13 |
| KS | 100 | pH | 0.66* | 0.49* | 0.03* | 1.42* | 0.58* | 0.56* | 0.00* | 1.24* |
| CLHS | 100 | pH | 0.66 ± 0.07 | 0.51 ± 0.07 | 0.03 ± 0.05 | 1.38 ± 0.17 | 0.63 ± 0.07 | 0.53 ± 0.06 | 0.02 ± 0.06 | 1.33 ± 0.15 |
| KM | 100 | pH | 0.64 ± 0.06 | 0.54 ± 0.07 | 0.02 ± 0.07 | 1.30 ± 0.16 | 0.60 ± 0.10 | 0.56 ± 0.08 | 0.03 ± 0.07 | 1.27 ± 0.17 |
| Random | 150 | pH | 0.68 ± 0.05 | 0.49 ± 0.04 | 0.01 ± 0.04 | 1.44 ± 0.12 | 0.68 ± 0.05 | 0.48 ± 0.04 | 0.04 ± 0.04 | 1.45 ± 0.13 |
| KS | 150 | pH | 0.73* | 0.45* | 0.04* | 1.56* | 0.64* | 0.50* | 0.02* | 1.39* |
| CLHS | 150 | pH | 0.71 ± 0.06 | 0.47 ± 0.06 | 0.02 ± 0.06 | 1.51 ± 0.15 | 0.69 ± 0.04 | 0.48 ± 0.04 | 0.03 ± 0.05 | 1.47 ± 0.11 |
| KM | 150 | pH | 0.65 ± 0.08 | 0.52 ± 0.07 | 0.00 ± 0.06 | 1.36 ± 0.18 | 0.60 ± 0.10 | 0.56 ± 0.09 | 0.03 ± 0.07 | 1.26 ± 0.19 |
| Random | 200 | pH | 0.72 ± 0.03 | 0.45 ± 0.03 | 0.01 ± 0.03 | 1.55 ± 0.09 | 0.72 ± 0.04 | 0.45 ± 0.04 | 0.04 ± 0.04 | 1.55 ± 0.13 |
| KS | 200 | pH | 0.76* | 0.42* | 0.04* | 1.66* | 0.73* | 0.45* | 0.07* | 1.55* |
| CLHS | 200 | pH | 0.73 ± 0.03 | 0.45 ± 0.03 | 0.01 ± 0.03 | 1.56 ± 0.09 | 0.72 ± 0.04 | 0.46 ± 0.04 | 0.05 ± 0.04 | 1.53 ± 0.11 |
| KM | 200 | pH | 0.65 ± 0.07 | 0.53 ± 0.07 | -0.03 ± 0.05 | 1.34 ± 0.17 | 0.55 ± 0.18 | 0.64 ± 0.20 | 0.00 ± 0.08 | 1.19 ± 0.32 |
| Random | 50 | Cation Exchange Capacity | 0.62 ± 0.06 | 5.96 ± 0.68 | 0.54 ± 0.86 | 0.84 ± 0.09 | 0.59 ± 0.07 | 5.95 ± 0.58 | 0.80 ± 1.00 | 0.84 ± 0.07 |
| KS | 50 | Cation Exchange Capacity | 0.59* | 5.75* | 0.66* | 0.86* | 0.65* | 5.31* | -0.13* | 0.93* |
| CLHS | 50 | Cation Exchange Capacity | 0.61 ± 0.07 | 6.04 ± 0.89 | 0.65 ± 0.97 | 0.83 ± 0.10 | 0.62 ± 0.05 | 5.72 ± 0.36 | 0.82 ± 0.86 | 0.87 ± 0.05 |
| KM | 50 | Cation Exchange Capacity | 0.62 ± 0.06 | 5.84 ± 0.63 | 0.64 ± 0.78 | 0.85 ± 0.09 | 0.60 ± 0.05 | 5.81 ± 0.44 | 0.80 ± 0.83 | 0.85 ± 0.06 |
| Random | 100 | Cation Exchange Capacity | 0.67 ± 0.04 | 5.56 ± 0.48 | 0.41 ± 0.49 | 0.89 ± 0.08 | 0.65 ± 0.05 | 5.41 ± 0.39 | 0.64 ± 0.64 | 0.92 ± 0.06 |
| KS | 100 | Cation Exchange Capacity | 0.68* | 5.13* | 0.80* | 0.96* | 0.69* | 5.11* | 0.78* | 0.97* |
| CLHS | 100 | Cation Exchange Capacity | 0.68 ± 0.04 | 5.42 ± 0.46 | 0.63 ± 0.56 | 0.92 ± 0.07 | 0.64 ± 0.08 | 5.59 ± 0.89 | 0.72 ± 0.84 | 0.90 ± 0.09 |
| KM | 100 | Cation Exchange Capacity | 0.67 ± 0.06 | 5.47 ± 0.81 | 0.28 ± 0.51 | 0.92 ± 0.11 | 0.65 ± 0.04 | 5.43 ± 0.36 | 0.54 ± 0.56 | 0.91 ± 0.06 |
| Random | 150 | Cation Exchange Capacity | 0.69 ± 0.03 | 5.37 ± 0.44 | 0.48 ± 0.39 | 0.92 ± 0.07 | 0.68 ± 0.05 | 5.19 ± 0.48 | 0.55 ± 0.45 | 0.96 ± 0.08 |
| KS | 150 | Cation Exchange Capacity | 0.70* | 5.20* | 1.01* | 0.95* | 0.64* | 5.49* | 0.83* | 0.90* |
| CLHS | 150 | Cation Exchange Capacity | 0.70 ± 0.03 | 5.28 ± 0.35 | 0.49 ± 0.42 | 0.94 ± 0.06 | 0.68 ± 0.04 | 5.14 ± 0.37 | 0.57 ± 0.47 | 0.97 ± 0.07 |
| KM | 150 | Cation Exchange Capacity | 0.66 ± 0.06 | 5.68 ± 0.78 | 0.38 ± 0.56 | 0.88 ± 0.11 | 0.64 ± 0.10 | 5.56 ± 0.99 | 0.57 ± 0.58 | 0.91 ± 0.13 |
| Random | 200 | Cation Exchange Capacity | 0.71 ± 0.02 | 5.17 ± 0.21 | 0.56 ± 0.24 | 0.96 ± 0.04 | 0.69 ± 0.03 | 5.08 ± 0.33 | 0.55 ± 0.36 | 0.98 ± 0.06 |
| KS | 200 | Cation Exchange Capacity | 0.73* | 4.78* | 0.54* | 1.03* | 0.61* | 5.71* | 0.28* | 0.86* |
| CLHS | 200 | Cation Exchange Capacity | 0.70 ± 0.02 | 5.26 ± 0.20 | 0.66 ± 0.21 | 0.94 ± 0.04 | 0.69 ± 0.04 | 5.04 ± 0.40 | 0.52 ± 0.32 | 0.99 ± 0.08 |
| KM | 200 | Cation Exchange Capacity | 0.65 ± 0.07 | 5.89 ± 0.88 | 0.57 ± 0.53 | 0.86 ± 0.12 | 0.60 ± 0.14 | 6.03 ± 1.52 | 0.61 ± 0.54 | 0.86 ± 0.16 |

*Only represents one repetition. RPIQ: Ratio of Performance to Interquartile distance, which can be calculated as RPIQ=IQ/RMSE where IQ = Q3-Q1; IQ being the interquartile distance of the validation set, Q1 the median of the first half of the validation set and Q3 the median for the second half of the validation set.
